# Construction of Skin‐Adaptable Slide‐Ring Hydrogels Based on Bile Acids Derived Polyrotaxanes for Smart Wound Dressing

**DOI:** 10.1002/advs.202520750

**Published:** 2025-12-27

**Authors:** Wen Huang, Xueru Xiong, Qian Sun, Xiangting Lai, Lili Cai, Yunhua Chen, Lin Wang, Yong‐Guang Jia

**Affiliations:** ^1^ School of Biomedical Science and Engineering South China University of Technology Guangzhou China; ^2^ School of Material Science and Engineering South China University of Technology Guangzhou China; ^3^ National Engineering Research Center for Tissue Restoration and Reconstruction South China University of Technology Guangzhou China; ^4^ School of Life Science Zhuhai College of Science and Technology Zhuhai China; ^5^ Center For Advanced Materials Research and Faculty of Arts and Sciences Beijing Normal University Zhuhai China

**Keywords:** bacteria killing, pulley effect, slide‐ring hydrogel, wound dressing, wound monitoring

## Abstract

Slide‐ring hydrogels (SR‐Gel) feature mechano‐adaptive networks with exceptional stretchability and skin‐adaptability, ideal for smart dressing applications. However, current SR‐Gel systems primarily rely on polyrotaxanes fabricated from poly(ethylene glycol) (PEG) and α‐cyclodextrins (α‐CDs), which typically possess an uncontrollable host‐guest coverage ratio. It is therefore crucial to develop efficient construction strategies that enable the fabrication of diverse SR‐Gels and broaden their scope and utility. Herein, we designed a polymerizable *pseudo*polyrotaxane with low host‐guest coverage via precise molecular recognition between β‐cyclodextrin (β‐CD) and a bile‐acid‐derived guest polymer. This precursor was photopolymerized with zwitterionic sulfobetaine methacrylate and N ‐ acryloyl glycinamide to construct a skin‐adaptable SR‐Gel. The sliding crosslinks provide enhanced chain mobility, endowing the hydrogel with high stretchability, excellent fatigue resistance, and the ability to adapt to wound deformation. Furthermore, levofloxacin‐loaded ZIF‐8 nanoparticles (ZIF‐8@Levo) were incorporated to obtain a conductive composite hydrogel (SR‐Gel/ZL), which was not only applied to treat bacterial infections, but could also stimulate and differentiate temperature, blood, and pressure, simultaneously. This work represents the first SR‐Gel system constructed from a bile acid/β‐CD polyrotaxane. Compared with the traditional PEG/α‐CD system, the host‐guest ratio can be precisely controlled, and the synthesis steps are simpler, providing a universal molecular model for the development of high‐performance slide‐ring materials.

## Graphical abstract

A novel β‐cyclodextrin/bile acid‐based slide‐ring hydrogel was prepared via precise host–guest recognition and photopolymerization. This smart hydrogel dressing exhibited skin‐adaptive mechanical properties, pH‐responsive antibacterial activity, and integrated multi‐signal sensing (temperature, blood, and pressure) for advanced wound management.

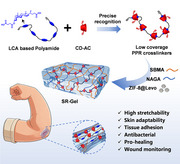

## 1 Introduction

Hydrogels have attracted extensive attention and gradually become considerable candidates for skin wound dressings because of their unique advantages, such as maintaining a moist environment on the wound surface, abundant functional groups, and excellent biocompatibility [1, 2, 3, 4]. However, compared with skin wounds in flat areas of the human body, the treatment of skin injuries in special areas, such as joints, necks, and muscle folds, is still challenging [5, 6]. The high mobility of the wound site and the inappropriate skin adaptability of the hydrogel dressing are the main limitations [7]. Moreover, promoting the wound healing process and effective wound management during the postwound closure time are also important to ensure an optimal healing status [8], since bacterial infection usually hinders the healing process [9]. Therefore, excellent skin‐adaptability, effective wound treatment, and timely monitoring of the wound status are essential functions for an ideal hydrogel dressing.

In recent decades, various approaches have been developed to improve the properties of hydrogels for wound dressings [10, 11, 12]. Typical examples include polyrotaxane‐based slide‐ring (SR) hydrogels, in which mobile cyclic molecules (hosts) tend to slide along linear polymers (guests) when stretched or compressed [13, 14, 15, 16]. Owing to the sliding crosslinks in the networks of SR hydrogels, a small number of polyrotaxane crosslinkers have shown obvious improvements in the stretchable mechanical abilities of materials [17, 18, 19, 20]. According to research by Ito et al., a high density of rings limits the range of chain sliding, and SR gels with 5% or lower host‐guest coverage have high extensibility [21, 22, 23, 24]. However, the existing SR gel systems primarily rely on α‐cyclodextrins (α‐CDs) as the ring components and polyethylene glycol (PEG) as the axle for construction [25, 26, 27], significantly limiting the diversity of SR gel systems. As a result, it is still a challenge to utilize the diverse range of guest polymers to build SR topological networks. Bile acids, a group of biocompatible guests, are unexplored for the construction of SR hydrogels. For example [28], bile acid and β‐cyclodextrin (β‐CD) self‐assembled into a complex via host‐guest recognition in the organic phase, which may be used to construct a skin‐adaptable SR gel system with an accurate host–guest coverage. The SR gel prepared by this system not only could retain the main advantages of traditional SR materials, such as high toughness and excellent fatigue resistance, but also significantly improve its biocompatibility and skin adaptability, due to the excellent biocompatibility of bile acid and β‐CD.

**SCHEME 1:**
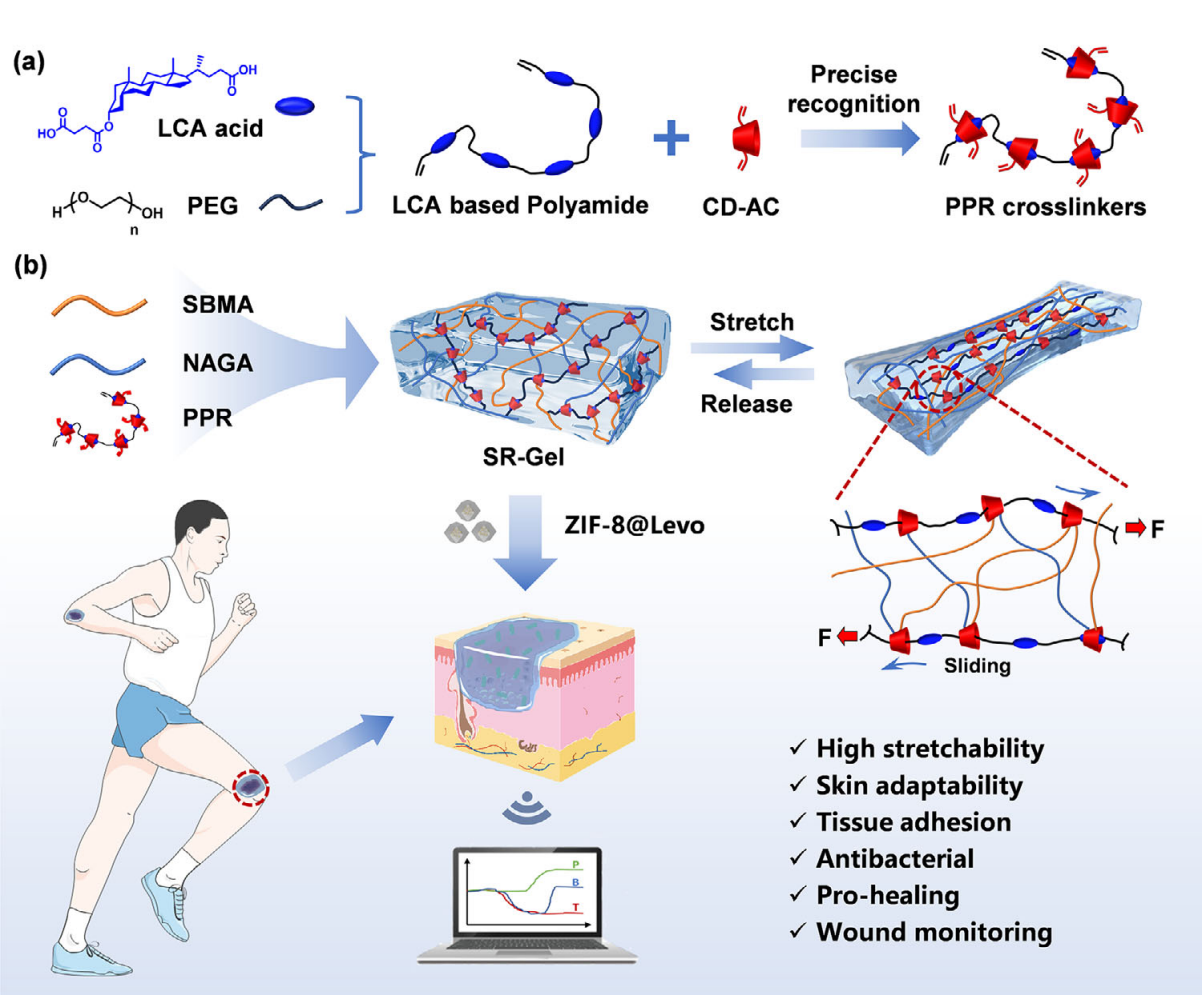
(a) Preparation of a polymerizable PPR crosslinker by threading CD‐Ac onto LP‐Ac composed of LCA and PEG segments via host‒guest recognition. (b) Design and network of SR‐Gel fabricated via SBMA, NAGA, and PPR crosslinkers, and (c) its applications in wound treatment and real‐time monitoring.

With the development of flexible electronic technology, wound dressings are needed to accurately reflect the healing status of wounds [29, 30]. Various smart wound dressings have been developed to monitor changes in relevant physiological and biochemical indicators at the wound sites [31, 32, 33]. For example, Zhang et al. [34] and Wang et al. [35] designed a resistance‐capacitance multimodal integrated hydrogel sensor that was applied in diabetic ulcer monitoring and respiratory monitoring, respectively. This model is an easy pathway for monitoring and distinguishing multiple signals in a single unit of wound dressing. In this work, bile acid‐β‐CD‐based SR hydrogels (SR‐Gel) were fabricated for wound treatment and real‐time monitoring. First, a polymerizable *pseudo*polyrotaxane (PPR) was prepared by threading acrylated β‐cyclodextrins (CD‐Ac) onto a linear lithocholic acid (LCA)‐based polyamide (LP‐Ac) via an accurate host‐guest recognition (Scheme  1a). The PPR was photocured with zwitterionic sulfobetaine methacrylate (SBMA) and N ‐ acryloyl glycinamide (NAGA) to realize a SR‐Gel system (Scheme 1b) in one step. The crosslink points could slide freely over a wide range, endowing the SR‐Gel with superior skin adaptability, such as extraordinary fatigue resistance and easy adaptation to the deformation and movement of the wound site without breaking, as well as repeatable tissue adhesion. Furthermore, antibacterial and conductive levofloxacin (Levo)‐loaded zeolitic imidazolate framework‐8 (ZIF‐8@Levo, ZL) nanoparticles were introduced into SR‐Gel to obtain SR‐Gel/ZL for constructing resistance‐capacitance integrated hydrogel sensors to continuously monitor key indicators of temperature, bleeding, and pressure, simultaneously (Scheme 1c). This work represents the first exploration of the application of SR materials in the field of smart dressings, demonstrating their great potential in the clinic.

## 2 Results and Discussion

### 2.1 Preparation of the PPR Crosslinker and SR‐Gel

**FIGURE 1:**
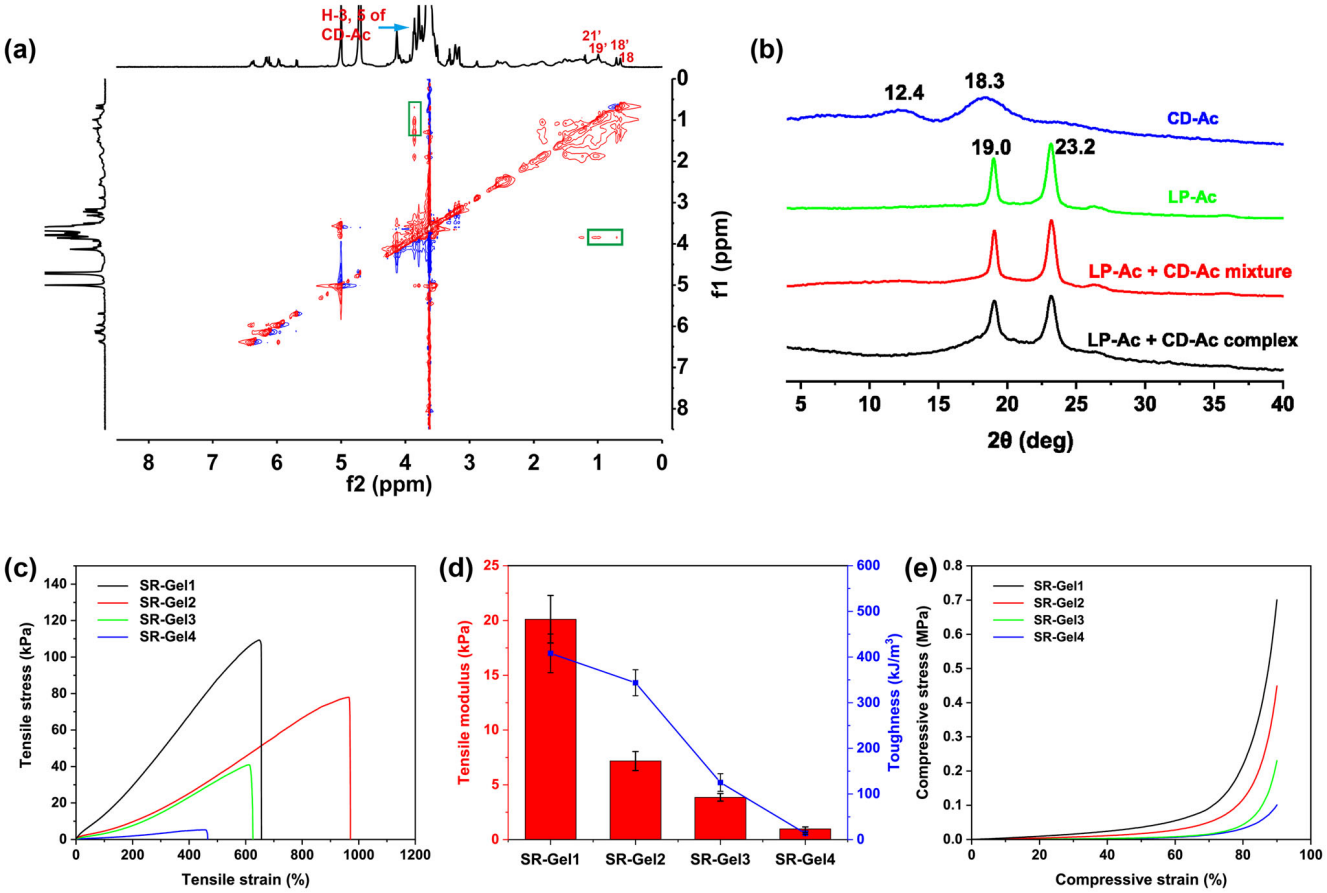
(a) 2D NOESY ^1^H NMR spectrum of the host‐guest complex of LP‐Ac with CD‐Ac in D_2_O (1/1 mol, 25°C). (b) Powder XRD patterns of LP‐Ac, CD‐Ac, and a mixture of LP‐Ac with CD‐Ac (1/1 mol) and the LP‐Ac/CD‐Ac host‐guest complex (1/1 mol). (c) tensile stress‐strain curves, (d) tensile modulus‐toughness curves and (e) compression curves of SR‐Gel. Values are expressed as the means ± SD (*n* = 3).

The PPR crosslinker was assembled via a precise host‐guest recognition of acrylated β‐CD with LCA‐derived units of linear polyamides, as shown in Scheme 1a. First, linear polyamides (PA‐LP) derived from LCA and PEG were prepared as guest molecules, and then PA‐LP and the host molecule β‐CD were both modified with acryloyl chloride to obtain LP‐Ac and CD‐Ac (Figures S1–S4), respectively, enabling PPR polymerizability and crosslinking. Host‐guest recognition in aqueous solution can be observed in the ^1^H NMR spectra. Figure S5 shows that the peaks of the three methyl protons of the LCA units all shift downfield (from peaks 18, 19, and 21 to peaks 18’, 19’, and 21’) upon the addition of CD‐Ac. Nuclear Overhauser enhancement spectroscopy (NOESY) was used to further investigate such ^1^H NMR spectral changes. The NOE correlation signals were attributed to peaks 18’ and 21’ with the interior H3/H5 of CD‐Ac (green rectangles in Figure 1a), which indicated that β‐CD slid over the PEG segments driven by host‒guest interactions toward the LCA units to form a PPR crosslinker. In addition, the solid‐state structure of the PPR crosslinker was investigated via powder X‐ray diffraction (XRD), as shown in Figure 1b, where two broad peaks (2θ) at 12.4 and 18.3° were assigned to the diffraction peaks of CD‐Ac, and the two characteristic diffraction peaks (2θ) at 19.0 and 23.3° were from the PEG segments of PPR. Compared with those of the mixtures of LP‐Ac and CD‐Ac, the two diffraction peaks of the PEG segments in the PPR broadened, indicating that complexation hindered the crystallization and decreased the crystallinity of the PEG segments, which was consistent with the lower *T_m_* in the DSC results (from 51.5 to 48.3°C, Figure S6). Owing to a more favorable and better fit complexation between β‐CD and bile acid units, CD‐Ac exclusively recognized the LCA units of LP‐Ac, and PEG segments could serve as the interval length of adjacent β‐CD units on LP‐Ac. In the polyrotaxane of α‐CD and PEG, a single α‐CD cavity would capture two ethylene glycol units of PEG segments [36, 37]. Similarly, the obtained PPR of LP‐Ac and CD‐Ac had a precise and low coverage at 2/45, ca. 4.44% (the number of ethylene glycol units in PEG with an average M_n_ of 2000 is approximately 45).

The obtained PPR crosslinker had good water solubility (ca. 88 g/L) and was convenient for allowing the PPR to directly crosslink the hydrophilic network in water to prepare hydrogels. SBMA has several advantages, including excellent biocompatibility, antifouling ability, and low immunogenicity, and has been widely used in wound dressings [32], surface coatings [38] and biomedical sensors [39]. NAGA is a fascinating monomer for high‐strength multifunctional hydrogel preparation because of its double amide hydrogen bond reinforcing mechanism [40]. The PPR crosslinker was photocured with SBMA and NAGA in a water system to directly obtain the multifunctional SR‐Gel (Table 1), in which the topological polyrotaxane was formed after end‐capped crosslinking of PPR (Scheme 1b). The hybrid SR‐Gel was confirmed by the FTIR spectra (Figure S8). The amide‐I/amide‐II vibrations were 1643/1551 cm^−1^ for PNAGA [41], and the absorption bands at 1023 cm^−1^ corresponded to sulfur‐SO_3_^−^ stretching vibrations, those at 1172 cm^−1^ corresponded to the quaternary ammonium group‐N^+^R_3_, and those at 1730 cm^−1^ were related to the ester carbonyl group C = O of PSBMA [42]. Both characteristic absorption bands were observed, indicating the successful incorporation of PPR, SBMA, and NAGA to form the SR‐Gel.

**TABLE 1:** The mass composition of each SR‐Gels.

Sample	Concentration (w/v%)			
	SBMA	NAGA	PPR	I2959
SR‐Gel1	25	35	0.6	0.5
SR‐Gel2	30	30	0.6	0.5
SR‐Gel3	35	25	0.6	0.5
SR‐Gel4	40	20	0.6	0.5

### 2.2 Mechanical Properties of the SR‐Gel

The double hydrogen bonds of NAGA, strong dipole interactions of SBMA, and topological crosslinking of SR constituted the network of SR‐Gel. First, the total monomer concentration was fixed at 60 w/v%, and the effects of the SR‐Gel composition on the mechanical properties were evaluated via tensile stress‐strain tests. As shown in Figure 1c–e, the tensile strength, tensile modulus, and toughness of the SR‐Gel constantly decreased with increasing SBMA content from 25 to 40 w/v%, which was attributed to the reduction in the number of double hydrogen bonds provided by the NAGA, weakening the tight interactions between the molecular chains. SR‐Gels with different compositions maintained excellent mechanical properties, with Young's modulus ranging from 0.97 ± 0.19 to 20.11 ± 2.17 kPa, elongations ranging from 471.57 ± 12.00 to 928.17 ± 37.60%, and tensile strengths ranging from 6.06 ± 1.30 to 107.06 ± 2.04 kPa; these mechanical properties highly matched those of human skin (Young's modulus ranging from 1 to 10 kPa [43]). The content of the PPR crosslinker was subsequently optimized (Figure S10). The obtained SR‐Gel2 with a monomer SBMA and NAGA ratio of 1:1 and a PPR crosslinker content of 0.6 w/v% could be stretched to more than 9 times its original length without breaking, allowing the SR‐Gel to deform to cover irregular wounds and adapt to skin deformation without breaking as a wound dressing, which was selected for subsequent experiments.

**FIGURE 2:**
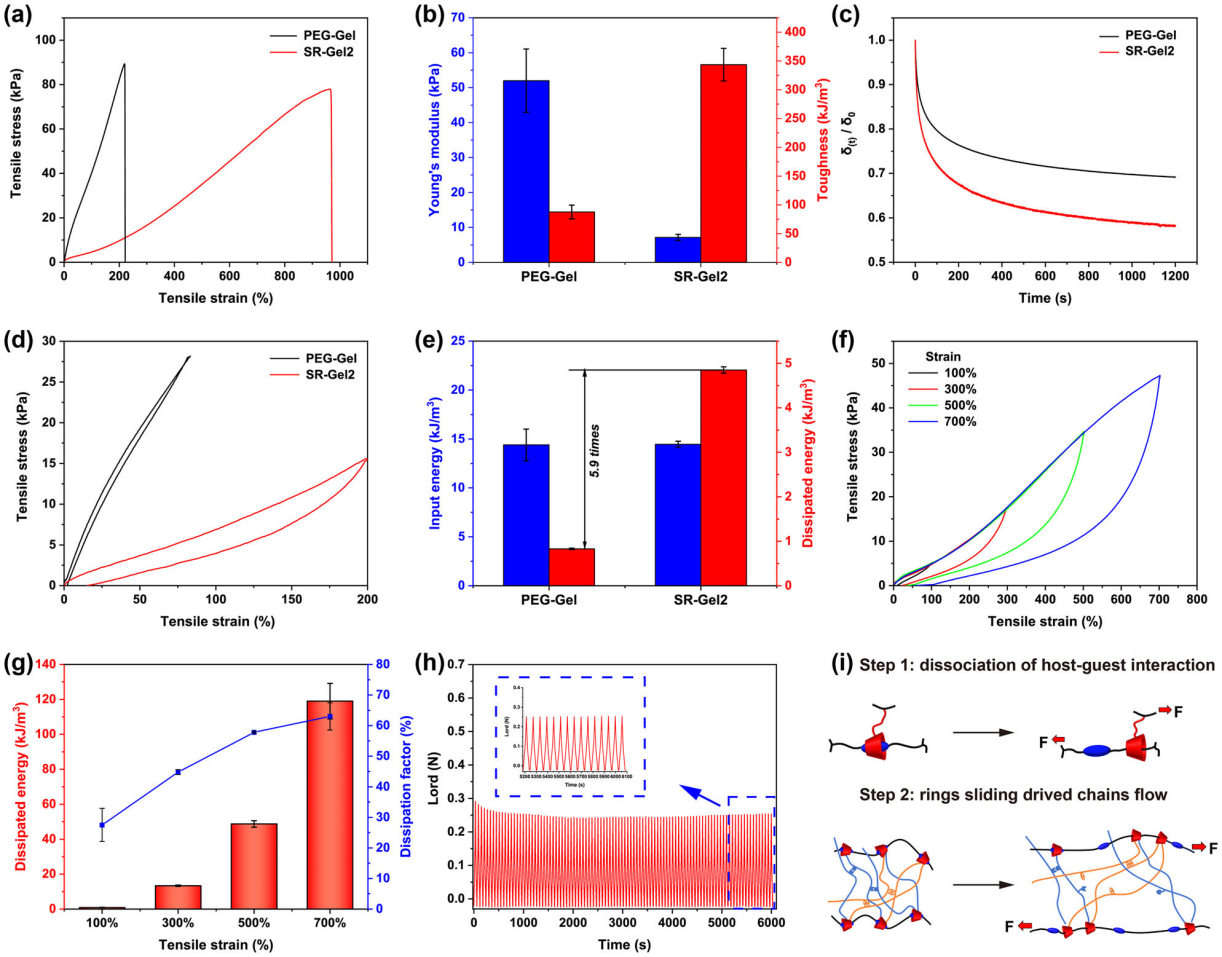
(a) Tensile stress‐strain curves of PEG‐Gel and SR‐Gel2. (b) Young's modulus and toughness of PEG‐Gel and SR‐Gel2. (c) The ratio of the residual stress δ to the initial stress δ_0_ for PEG‐Gel and SR‐Gel2 as a function of time during the relaxation process at a pre‐stretched strain of 180%. (d) Single tensile loading‐unloading cycle curves and (e) input energy and dissipated energy of PEG‐Gel and SR‐Gel2. (f) Tensile loading‐unloading cycle curves and (g) dissipated energy and dissipation factors of SR‐Gel2 recorded with increasing maximum strain. (h) Load‐time curve of SR‐Gel2 at a strain of 200% over 100 continuous cycles (the curves in the blue rectangle correspond to the last fifteen cycles). (i) Schematic diagrams of the toughening mechanisms of SR‐Gel. Values are expressed as the means ± SD (*n*  =  3).

For comparison, a hydrogel crosslinked with PEG diacrylate (average M_n_ 1000) was prepared and denoted as PEG‐Gel to reveal the contribution of the SR topological network to the properties of the SR‐Gel. Figure 2a,b confirmed that SR‐Gel2 performed much better than PEG‐Gel in terms of stretchability (928.17 ± 37.60 vs 217.61 ± 22.04%) and toughness (343.56 ± 28.40 vs 87.59 ± 11.93 kJ/m^3^) at the same concentration of crosslinkers but presented a lower Young's modulus (7.17 ± 0.87 vs 52.00 ± 9.07 kPa). Compared with PEG‐Gel, the SR‐Gel exhibited a significant deformability increase and unusual stiffness‐toughness independence in fixed crosslinked networks, and this discrepancy was clearly attributed to the free movement of crosslinking points over a wide range in the SR system. To understand the sliding motions of the SR units in SR‐Gel, stress‐relaxation experiments were performed, as shown in Figure 2c. Under the same pre‐strain, SR‐Gel2 relaxed stress much faster and ultimately maintained less residual stress than did PEG‐Gel. We speculated that the free sliding motions of the CDs on the PEG chains resulted in a much looser network of SR‐Gel, thus resulting in faster and more adequate relaxation [44]. The rheological properties of the hydrogels were tested later. As shown in Figure S11a,b, SR‐Gel2 underwent a gel‐sol transition (the loss modulus G′ was equal to the storage modulus G″) at a lower frequency and strain than did PEG‐Gel, confirming that the greater fluidity inside the SR‐Gel2 network was attributed to the sliding movement of cyclic crosslinks along the linear polymer chains. Moreover, as shown in Figure S11c, the ratio of G′ to G″ (tan δ value) of SR‐Gel2 was considerably greater than that of PEG‐Gel, suggesting a newly efficient energy dissipation mechanism related to the topological structure of the SR‐Gel system [45].

These unique dynamic characteristics of the SR‐Gel were also evaluated via cyclic tensile measurements (Figure 2d). Keeping a similar input energy density (∼14.4 kJ/m^3^), the hysteresis area between the loading and unloading curves of SR‐Gel2 was much larger than that of PEG‐Gel. Accordingly, the energy dissipation density of SR‐Gel2 was calculated to be 4.85 ± 0.072 kJ/m^3^, which was approximately 5.9 times greater than that of PEG‐Gel (Figure 2e), confirming the “pulley effect” of the SR structure in SR‐Gel2. Furthermore, progressive cyclic tensile experiments were performed to trace the changes in energy dissipation under different applied strains (Figure 2f,g). The energy dissipation performance can also be evaluated by the damping capacity, which is defined as the ratio of the dissipated energy to the input energy. The damping capacity increased from 27.51 ± 5.41% to 63.03 ± 4.45% with increasing strain applied to SR‐Gel2. These results revealed that multiple toughening mechanisms allowed the SR‐Gel to exhibit exceptional mechanical properties (Figure 2i). First, the “pulley effect” dispersed the stresses of the system and reduced the damage caused by stress concentrations to the network. The key to this mechanism was that the low coverage provided enough sliding space for CDs [46, 47]. Subsequently, more external energy was used to overcome the host‐guest dissociation of LCA and CDs as well as the plastic flow of the polymer chain driven by ring sliding, which represented a continuous energy dissipation mechanism that provided an excellent complement to other energy dissipation modes that usually rely on sacrificial bond breaking only [48, 49]. The fatigue resistance of the SR‐Gel was also investigated. SR‐Gel2 could undergo 100 successive stretch‐relaxation cycles at a strain of 200%, with the maximum force decreasing slightly after a few cycles and then remaining at 87.06% of that of the first cycle (Figure 2h). This long‐lasting durability is necessary to accommodate continuous skin movement and extend the service life of wound dressings.

### 2.3 Adhesion Properties of SR‐Gel

**FIGURE 3:**
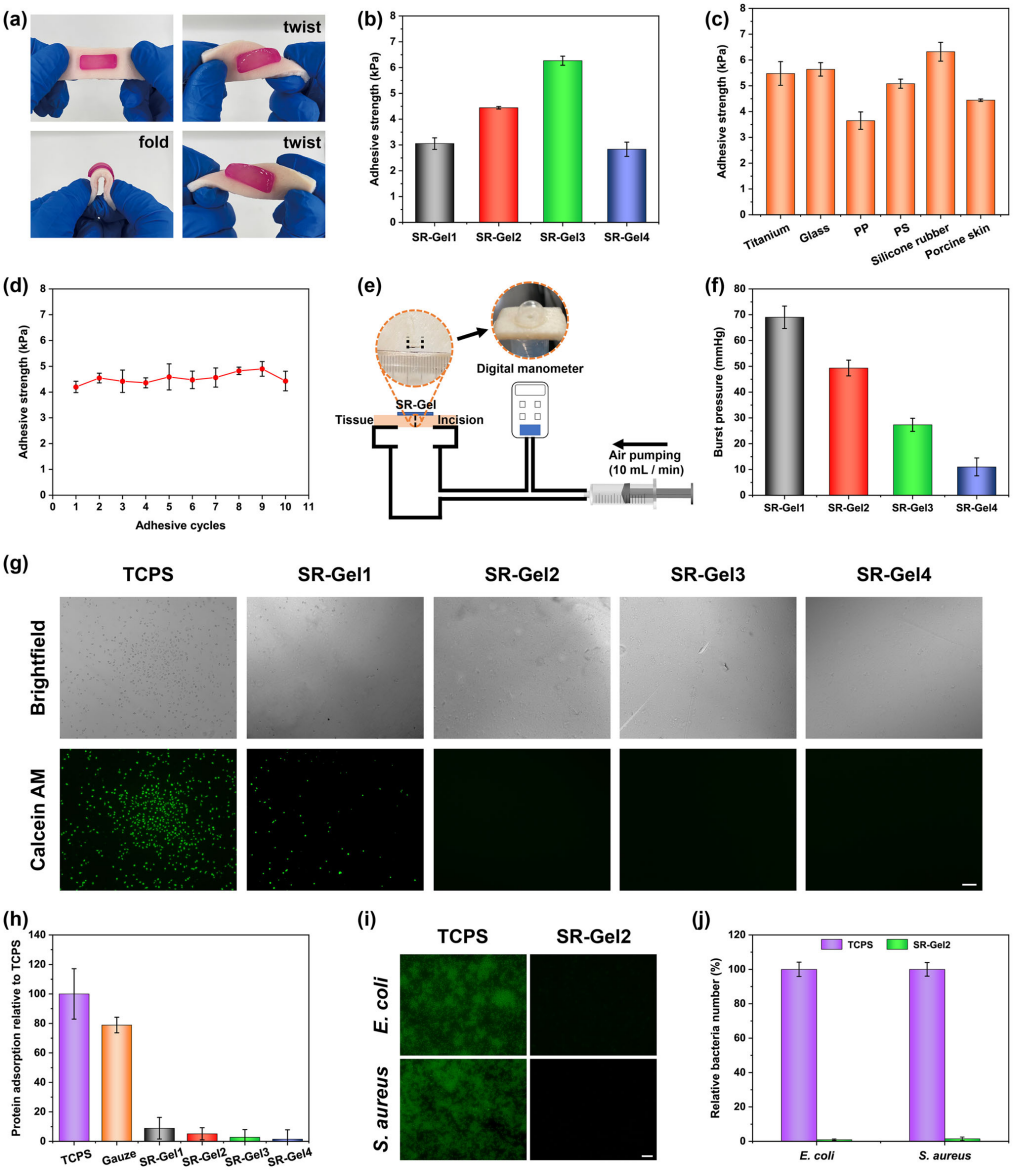
(a) SR‐Gel2 adhering to pigskin underwent different types of deformation. (b) Adhesive strength of different SR‐Gels adhering to pigskin. (c) Adhesive strength of SR‐Gel2 adhering to various substrate surfaces. (d) Adhesive strength of SR‐Gel2 after 10 peeling‐adhesion cycles. (e) Schematic diagram of the burst pressure test of SR‐Gel. (f) Burst pressure of SR‐Gel2. (g) Brightfield micrographs and fluorescence micrographs of L929 cells attached to the TCPS and SR‐Gel after coculture for 24 h (the live cells were treated with calcein AM and subjected to green staining; scale bar, 100 µm). (h) Protein adsorption of SR‐Gel; Tissue culture polystyrene (TCPS) and gauze were used as the control groups. (i) Fluorescence micrographs of *E. coli* and *S. aureus* attached to TCPS and SR‐Gel2 after coculture for 24 h (the live bacteria were treated with FDA and subjected to greening staining; scale bar, 20 µm). (j) The relative bacterial number. Values are expressed as the means ± SD (*n*  =  3).

Dressings with tissue adhesive could adhere to skin wounds without the need for other tapes to ensure a favorable healing environment. The NAGA chains have many amide groups, which easily form multiple hydrogen bonds with polar groups [50]. Moreover, the anionic and cationic groups of zwitterions are known to exhibit strong electrostatic interactions with amino, carboxyl, and amide groups on tissue surfaces (Figure S12a) [51, 52]. Thus, the synergistic effect of electrostatic interactions and hydrogen bonding enabled SR‐Gel2 to adhere appropriately to various tissues (heart, gizzard, intestine, liver, lung, and skin, Figure S12b) and substrates (glass, metal, ceramics, quartz, and rubber, Figure S12c). As shown in Figure 3a, pigskin with SR‐Gel2 attached was folded and twisted to simulate the movement of different parts of the human body, such as knuckles, elbows, and knees. The hydrogel remained firmly adhered to the pigskin without breaking or debonding. Additionally, as the degree of finger flexion increased from 0° to 135°, SR‐Gel2 could adhere tightly to the knuckle without buckling, resulting in excellent tissue adhesion and skin adaptability (Figure S12d). Figure 3b showed that the adhesive strength of SR‐Gel increased with increasing SBMA content, and the adhesive strength reached 2.83 ± 0.28 to 6.27 ± 0.18 kPa. SR‐Gel also had an appropriate adhesion strength to various substrate surfaces (Figure 3c). The cyclic peeling‐adhesion tests also revealed that the adhesive strength of SR‐Gel2 essentially remained stable after 10 peeling‐adhesion cycles (Figure 3d). Furthermore, burst pressure tests using pigskin as the substrate were performed to further quantify the adhesion of SR‐Gel2 (Figure 3e). As shown in Figure 3f, the SR‐Gels with various compositions presented the highest burst pressure of 69.00 ± 4.36 mmHg, demonstrating the ability of the SR‐Gels to seal the wounds. These results suggested that the SR‐Gel had excellent, stable, and recyclable adhesive strength and could firmly adhere to the wound surface and adapt to the deformation of the skin, which is the basic performance of hydrogel wound dressings or biosensors.

### 2.4 In Vitro Antibiofouling Performance

The harmful adhesion of microorganisms, such as bacteria and cells, is a common challenge in wound dressings, medical implants, biosensors, and other fields [53, 54]. Zwitterions have strong surface hydration and hydrophilicity, can resist the bioadhesion of cells, proteins, bacteria, etc., and have been widely used in the field of antifouling materials [55]. We measured the water contact angle of SR‐Gel using a contact angle goniometer, Figure S13 showed that SR‐Gels had excellent hydrophilicity, and as the SBMA content increased, the contact angle of SR‐Gel decreased from 18.7 ± 2.1° to 9.6 ± 1.4°. Herein, SR‐Gel was exposed to cells, proteins, and bacteria to evaluate its antibiofouling properties. Tissue culture polystyrene (TCPS) and gauze were used as controls. As shown in Figure 3g, after 24 h of coculture, the L929 cells in the SR‐Gel groups were much more resistant to adhesion than were those in the TCPS control group. In detail, many L929 cells with flat and extended shapes adhered well to the TCPS, while only a few floating rounded cells were present on SR‐Gel1, and almost no cell adhesion was observed in the other groups. Moreover, we evaluated the antifouling ability of SR‐Gel by detecting protein adsorption. As shown in Figure 3h, the SR‐Gels with various SBMA contents presented lower protein adsorption (from 1.49 ± 6.34% to 8.89 ± 7.33%), while the corresponding TCPS and gauze samples presented greater protein adsorption (100.00 ± 17.09% and 78.90 ± 5.25%, respectively). These results suggested that the introduction of SBMA strengthened the anti‐fouling performance of SR‐Gel.

Blood contamination is a major challenge for traditional dressings, and frequent dressing changes are not conducive to wound healing and affect their practical convenience. Thus, the antifouling ability of SR‐Gel2 against whole blood was shown in Figure S14. Traditional dressings and SR‐Gel‐coated dressings were incubated in whole blood for 2 h at 37°C. Traditional dressings are heavily contaminated by whole blood, whereas antibiofouling SR‐Gel2 coatings resist most of the blood contamination of dressings. In addition, the antibacterial fouling ability of SR‐Gel2 against *E. col*i and *S. aureus* was investigated. As shown in Figure 3i, after coculture with *E. coli* and *S. aureus* for 24 h, many live bacteria were clearly observed on the TCPS, whereas minimal bacterial adhesion was observed on SR‐Gel2. Compared with TCPS, only 0.98 ± 0.43% of *E. coli* and 1.50 ± 1.00% of *S. aureus* adhered to the surface of SR‐Gel2 (Figure 3j), which indicated that SR‐Gel could isolate bacteria and wounds and prevent bacterial colonization to reduce the occurrence of wound infections.

### 2.5 Antibacterial Properties and Biocompatibility In Vitro

**FIGURE 4:**
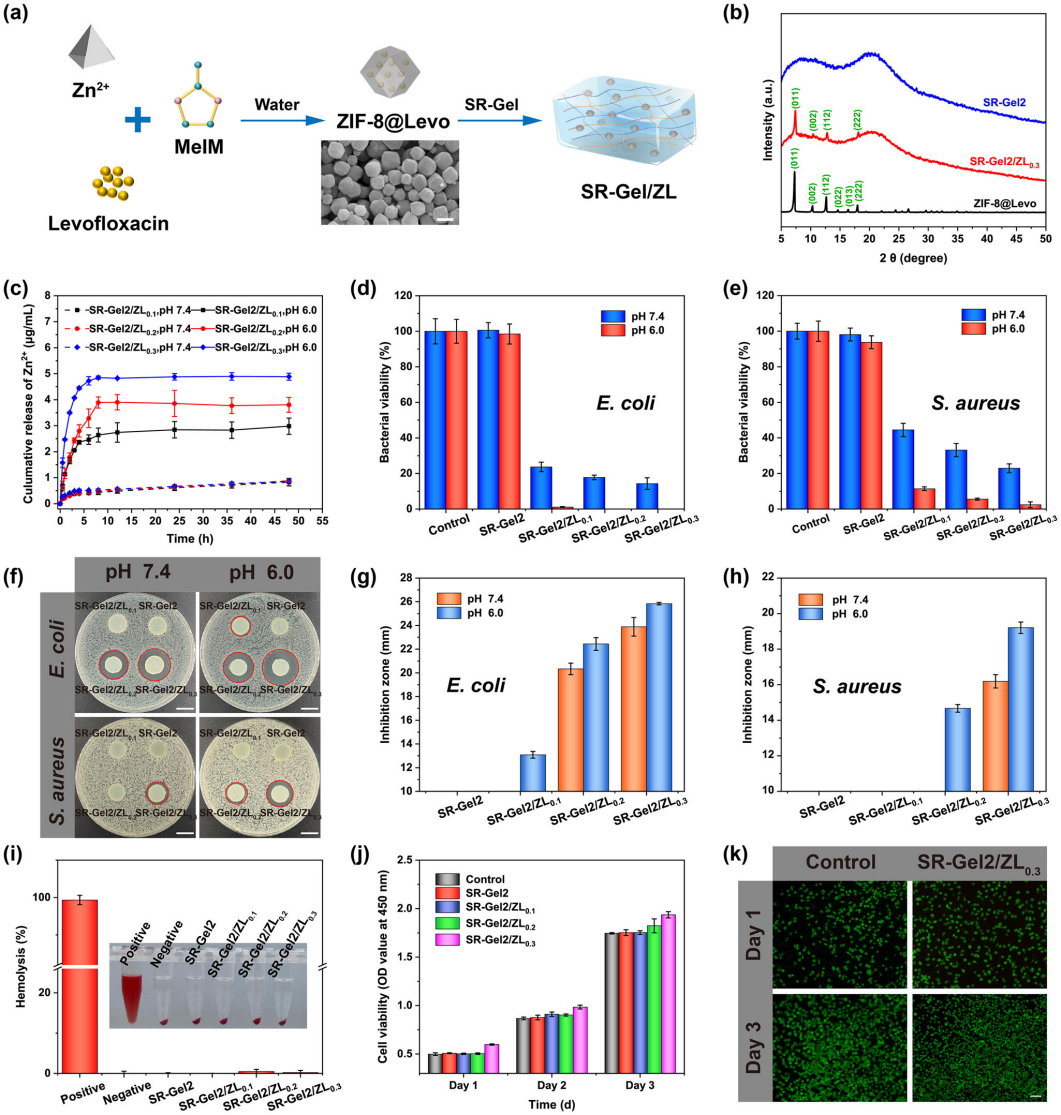
(a) Schematic diagram of the preparation of the SR‐Gel/ZL hydrogel and SEM image of the ZIF‐8@Levo nanoparticles (scale bar, 500 nm). (b) Powder XRD patterns of ZIF‐8@Levo, SR‐Gel2 and SR‐Gel2/ZL_0.3_ (the ZIF‐8@Levo concentration was 0.3 w/v%). (c) Zn^2+^ release profiles of SR‐Gel/ZL in PBS buffer at pH 7.4 and 6.0. Bacterial viability of (d) *E. coli* and (e) *S. aureus* after treatment with hydrogels at pH 7.4 and 6.0. (f) Optical images and plots of the inhibition zones of the hydrogels against (g) *E. coli* and (h) *S. aureus* at pH 7.4 and 6.0 (scale bar, 10 mm). (i) Optical image and plot of hemolysis of hydrogels. (j) Cell viability of L929 cells after incubation with the extracts of the hydrogels (extracting for 48 h), as determined by a CCK‐8 assay. (k) Live/dead staining of L929 cells after incubation with the SR‐Gel2/ZL_0.3_ extracts for 1and 3 days (scale bar, 100 µm). Values are expressed as the means ± SD (*n*  =  3).

The microenvironment of wounds usually becomes acidic due to bacterial metabolites during infection, which has inspired the design of many acid‐sensitive antimicrobial materials [56]. For example, zeolitic imidazolate framework‐8 (ZIF‐8) is a zinc‐based metal‐organic framework whose structure is easily degraded in acidic environments and is therefore often used as a pH‐sensitive drug delivery system [57, 58, 59]. Here, levofloxacin‐loaded ZIF‐8 nanoparticles (ZIF‐8@Levo) were designed and introduced into SR‐Gel2 to prepare multifunctional composite hydrogel dressings, named SR‐Gel2/ZL_x_ (x represents the concentration of ZIF‐8@Levo) (Figure 4a). As shown in Figure S15a–e, scanning electron microscopy (SEM) images and powder XRD patterns of ZIF‐8 and ZIF‐8@Levo revealed that these two nanoparticles were highly crystalline and composed of a uniform rhombic dodecahedral morphology. As shown in Figure S15f,g, all the characteristic peaks related to ZIF‐8 and the drug were observed in ZIF‐8@Levo, indicating that levofloxacin had been embedded inside the ZIF‐8 crystals (the drug loading effifiency (DLE) and entrapment efficiency (EE) of levofloxacin in ZIF‐8@Levo was 3.52% and 7.86%). Figure 4b showed that some sharp diffraction peaks of ZIF‐8@Levo appeared in SR‐Gel2/ZL_0.3_, revealing the successful introduction of nanoparticles into the SR‐Gel system. Besides, we compared the mechanical properties of SR‐Gel2 and SR‐Gel2/ZL0.3 via tensile tests. Figure S16 showed that the incorporation of ZIF‐8@Levo nanoparticles did not impair the mechanical properties of SR‐Gel2. Instead, it slightly improved tensile modulus, tensile strength, and toughness, while maintaining stretchability that matched skin movement. This was because ZIF‐8@Levo nanoparticles were uniformly dispersed in the hydrogel network and did not disrupt the slide‐ring crosslinking structure.

Figure 4c and Figure S18 showed the pH‐responsive cumulative release profiles of Zn^2+^ and levofloxacin at pH 7.4 and 6.0, respectively. The PBS buffer (pH 7.4) caused minimal Zn^2+^ and levofloxacin release, whereas the acidic environment (pH 6.0) stimulated rapid release of Zn^2+^ and levofloxacin, which demonstrated that SR‐Gel/ZL was a promising and effective drug delivery system for controlling Zn^2+^ and levofloxacin release in the acid‐infected microenvironment. First, we tested the antibacterial ratios of SR‐Gel/ZL via the spread plate method against the gram‐negative bacteria *E. coli* and the gram‐positive bacteria *S. aureus*, and the results are shown in Figure 4d,e. The antibacterial performance of SR‐Gel/ZL significantly increased with increasing ZIF‐8@Levo nanoparticle content, and the antibacterial capacity of SR‐Gel/ZL at pH 6.0 was further enhanced compared with that at pH 7.4, in which SR‐Gel/ZL_0.3_ containing 0.3 w/v% nanoparticles had the highest antibacterial activity. Specifically, the antibacterial ratio of SR‐Gel2/ZL_0.3_ increased to approximately 100% against *E. coli* and 97.58 ± 1.59% against *S. aureus*. Next, the inhibition zone method was adopted to further evaluate the antibacterial activity of the hydrogels. Figure 4f–h show that the concentrations of Zn^2+^ and levofloxacin diffused on the agar plates were not enough to inhibit the growth of bacteria, and no significant inhibition zones appeared. SR‐Gel/ZL was able to increase drug release at pH 6.0, resulting in obviously larger inhibition zones for SR‐Gel/ZL in acidic environments than for the groups at pH 7.4. These results suggested that SR‐Gel/ZL was able to enhance the antibacterial properties through the release of more drugs under acidic conditions. This acid‐stimulated antibacterial performance is beneficial for the healing of infected wounds.

A hemolysis test was performed to evaluate the blood compatibility of the SR‐Gel. As shown in Figure 4i, the hemolysis ratio of each SR‐Gel group was very low (<0.5%), whereas the positive control group was bright red in color with an extremely high hemolysis ratio. The cytotoxicity of the SR‐Gel was tested by culturing L929 cells with hydrogel extracts (extracting for 48 h). The results of the CCK‐8 and live/dead assays are shown in Figure 4j,k and Figure S19, respectively. The number of cells that proliferated over time increased, and very few dead cells were observed in the fluorescence images. The cell viabilities of the SR‐Gel, SR‐Gel/ZL_0.1,_ and SR‐Gel/ZL_0.2_ groups were comparable to that of the control group after being cultured for three consecutive days. Even with SR‐Gel/ZL_0.3_, the cell viability was slightly greater than that of the control, which was attributed to the promoting effect of releasing an appropriate concentration of bioactive Zn^2+^ on cell proliferation [60, 61]. Therefore, all of the above results illustrated that the SR‐Gel/ZL hydrogel possessed good hemocompatibility and cytocompatibility.

### 2.6 In Vivo Wound Healing Assessment

**FIGURE 5:**
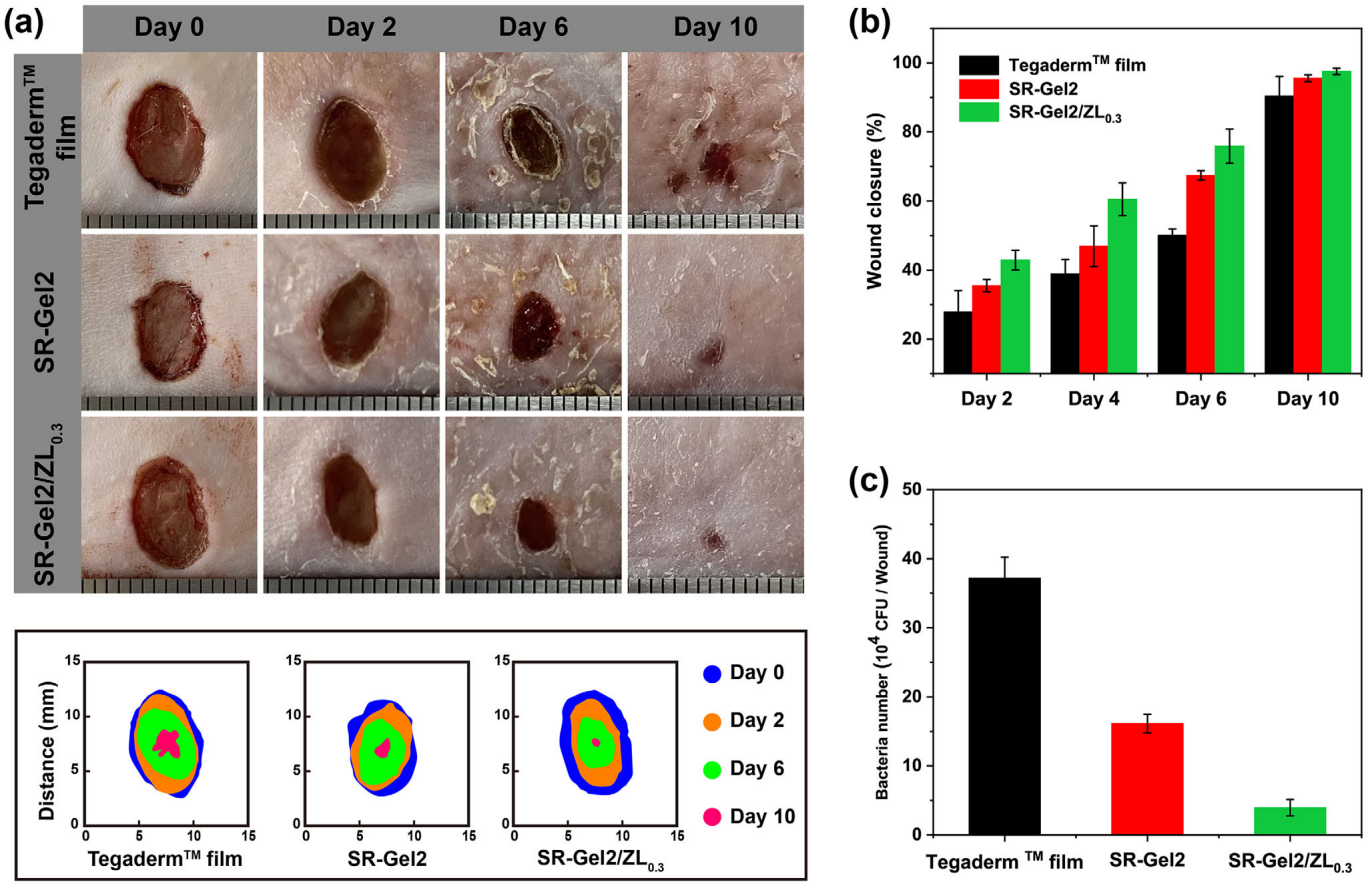
(a) Representative infected wound photographs and corresponding wound areas at days 0, 2, 6, and 10 in each group. (b) Wound closure of each group at defined time points. (c) The number of bacteria in infected tissues after treatment for 2 days. Values are expressed as the means ± SD (*n*  =  3).

An infected full‐thickness mouse skin defect model was generated to evaluate the actual ability of the SR‐Gel/ZL hydrogel to promote infected wound healing. SR‐Gel2/ZL_0.3_ was selected as the representative dressing material because of its excellent skin adaptability, optimal antibacterial activity, and biocompatibility. SR‐Gel2 without nanoparticles was used in another experimental group, and a commercially available Tegaderm film was chosen as a control. As shown in Figure 5a,b, after treatment for 2 days, an obvious decrease in the wound area was observed for the SR‐Gel2 and SR‐Gel2/ZL_0.3_ groups compared with the Tegaderm film group. Quantitative analysis of the wound areas revealed that after treatment for 4 days, the ratios of wound closure in SR‐Gel and SR‐Gel/ZL groups were 46.92 ± 5.91% and 60.54 ± 4.70%, respectively, whereas that in the Tegaderm film group was 38.97 ± 4.13%, revealing that SR‐Gel2/ZL_0.3_ had the best effect on wound healing. Moreover, after 10 days, the wounds in the Tegaderm film group were still unclosed, but the wounds in the SR‐Gel2/ZL_0.3_ group nearly completely healed and even contained smooth new epidermal tissue. Additionally, bacterial counting analysis of infected tissues in Figure 5c and Figure S20 revealed that the Tegaderm film group contained countless colonies on agar plates, whereas the SR‐Gel2/ZL_0.3_ treatment for 2 days reduced the number of bacteria in the wounds by approximately 10‐fold.

**FIGURE 6:**
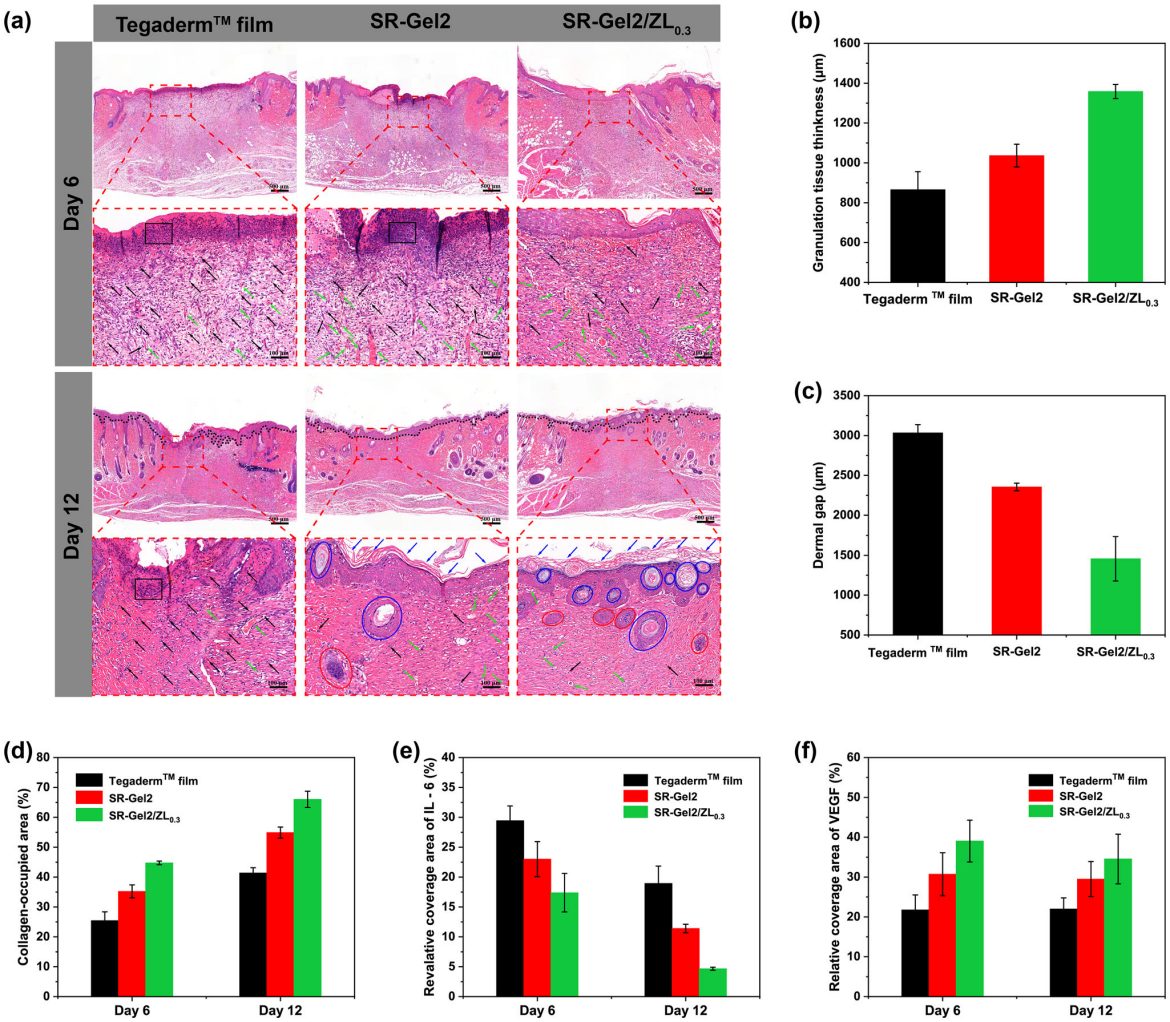
(a) H&E staining of tissues collected from the wound areas on the sixth and 12th days (scale bar, 100 µm; black box, cell debris; black dashed line, boundary of the epithelium and dermis; black arrow, inflammatory cells; green arrow, blood vessels; blue arrow, stratum corneum; red circle, sebaceous glands; blue circle, hair follicles). (b) Granulation tissue thickness and (c) dermal gap at the wound sites in each group on the sixth day. (d) Collagen‐occupied area of tissues collected from the wound areas on the sixth and 12th days. The quantified data of the relative area coverage of (e) IL‐6 and (f) VEGF. Values are expressed as the means ± SD (*n*  =  3).

Histological analysis was performed to further evaluate the quality of regenerated wound tissue after 6 and 12 days of hydrogel treatment. H&E staining results (Figure 6a) revealed many dead cell debris (black box) and inflammatory cell infiltration (black arrow) in the Tegaderm film and SR‐Gel groups due to severe infection on the sixth day, whereas the number of inflammatory cells was significantly reduced in the SR‐Gel2/ZL_0.3_ group, which was attributed to the excellent antibacterial properties of the SR‐Gel2/ZL_0.3_ hydrogel. A greater density of neovascularization was found in the hydrogel groups (green arrow), which was attributed to the upregulation of key genes associated with tube formation by SBMA and Zn^2+^ in the hydrogels [42, 61]. After 12 days of treatment, the wounds in the Tegaderm film group had not yet closed, whereas those in the other groups presented a greater degree of epithelialization, all with fully structured epithelium and stratum corneum, and even more sebaceous glands and hair follicles clearly appeared in the SR‐Gel2/ZL_0.3_ group. Furthermore, the granulation tissue thickness on the sixth day was marked, and the samples were counted. A significantly greater granulation tissue thickness and smaller dermal gap of SR‐Gel2/ZL_0.3_ were shown in Figure 6b,c, indicating that a faster wound regeneration rate was promoted by the SR‐Gel2/ZL_0.3_ hydrogels.

Collagen, a major component of the extracellular matrix, is also an important indicator of the wound healing process. Figure S21 showed the degree of collagen deposition (stained blue) in the regenerated skin as detected by Masson's trichrome staining. Compared with those in the other two groups, the wound area tissue in the SR‐Gel2/ZL_0.3_ group appeared to have a deeper blue color and better‐organized collagen fibers (green box), indicating higher collagen levels. The statistical results of the areas of blue in each group (Figure 6d) were consistent with the above observations. IL‐6 and VEGF are two important cytokines that are closely related to the state of wound healing and were selected as indicators to evaluate the effectiveness of hydrogels (Figure S22). Quantitative analysis of the area of wound immunohistochemical staining revealed that the expression of IL‐6 gradually decreased over time. Compared with the other two groups, the SR‐Gel2/ZL_0.3_ group presented the lowest expression of IL‐6 (Figure 6e), which was mainly because its excellent antibacterial ability reduced the degree of bacterial infection. Moreover, the SR‐Gel2/ZL_0.3_ group presented the highest VEGF expression level among the three groups (Figure 6f). These results all indicate that the hydrogel dressing of SR‐Gel/ZL is a promising candidate for treating bacterial infections and accelerating tissue repair during wound healing.

### 2.7 Multiple Sensing of SR‐Gel/ZL Sensor

**FIGURE 7:**
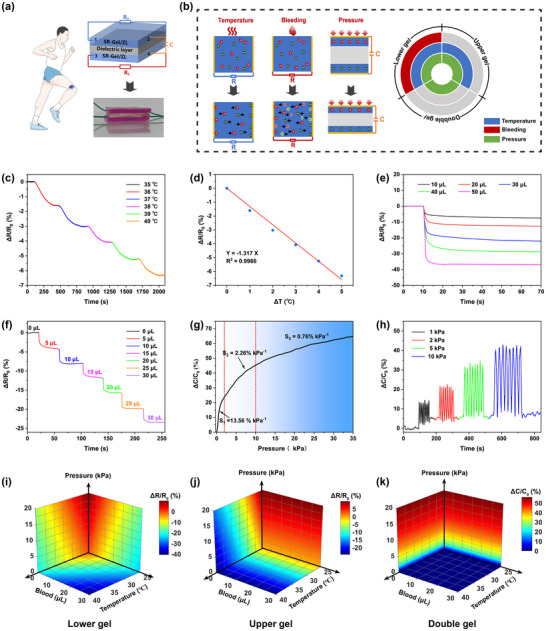
(a) Schematic design and simplified equivalent electrical circuits of this hydrogel sensor. (b) Sensing mechanism of the SR‐Gel/ZL‐based capacitive and resistive hydrogel sensors upon temperature, bleeding, and pressure stimulus. (c) Normalized relative resistance curve of SR‐Gel2/ZL_0.3_ with increasing temperature from 35 to 40°C. (d) Standard resistance curve of the temperature response. The normalized relative resistance of SR‐Gel2/ZL_0.3_ with blood volume changes from (e) 0.01 to 0.05 mL and (f) from 0 to 0.03 mL (each consecutive response to 5 µL of blood). (g) The normalized relative capacitance curve of double hydrogels with pressures ranging from 0 to 35 kPa. (h) The normalized relative capacitance of double hydrogels in response to a constant load. 3D colormaps of the resistance or capacitance of the (i) lower gel, (j) upper gel, and (k) double gel of the sandwich‐structured skin sensor.

The continuous real‐time monitoring of temperature, bleeding, and pressure at wound sites can effectively reflect the status of wounds, facilitating the management and treatment of wounds [62, 63], especially chronic or infected wounds. Unlike traditional dressings, the dressing based on the SR‐Gel/ZL hydrogel was equipped with ionic conductivity due to the presence of zwitterions and Zn^2+^ and exhibited excellent deformability, adhesion, and biocompatibility. To design a multifunctional hydrogel sensor, a sandwich structure was designed by sandwiching a commercial elastomer (VHB 4910, 3 M) as a dielectric layer between two SR‐Gel2/ZL_0.3_ hydrogel layers. Four metal electrodes were embedded in the hydrogel layers to create two resistance sensors and one capacitance sensor in a single unit (Figure 7a). Its working mechanism is displayed in Figure 7b. The lower resistance sensor responded to signal changes in wound temperature, bleeding, and pressure while also promoting wound healing; the upper resistance sensor responded to temperature and pressure changes; and the capacitance of the double hydrogel was affected only by pressure.

First, we tested the temperature sensing ability of SR‐Gel2/ZL_0.3_ in the range of 35–40°C to simulate the temperature of the wounds. The normalized relative resistance variation (*ΔR/R_0_*) was recorded to evaluate the temperature response (*ΔR*, the change in the instantaneous resistance under measured pressure; *R_0_*, the initial resistance). Figure 7c showed that when the temperature increased from 35 to 40°C, the relative resistance of SR‐Gel2/ZL_0.3_ decreased, which was mainly because heating accelerated the transport of ions in the hydrogel. The temperature was linearly related to *ΔR/R_0_* (R^2^> 0.99, Figure 7d), and a gauge factor (GF) of 1.32%/°C was achieved. Moreover, the SR‐Gel2/ZL_0.3_ sensor also responded well over a wide temperature range (from 25 to 65°C, Figure S23). Blood contains abundant ions (e.g., Na^+^, Cl^−^, K^+^, PO_4_^3−^); however, SR‐Gel2/ZL_0.3_ has excellent swelling properties that allow it to absorb blood and increase the concentration of conductive ions, resulting in a change in resistance. The SR‐Gel2/ZL_0.3_ skin sensor was sensitive to blood (Figure 7e,f), and its volume was linearly related to *ΔR/R_0_* (Figure S24). Notably, the sensor exhibited a fast response (<2 s) to blood with a GF of 0.71%/µL. The value of capacitance (*C*) can be calculated by the equation *C = εA/4πkd* (*d*, the distance between electrodes). When the SR‐Gel2/ZL_0.3_ sensor was compressed under pressure, the distance between the double hydrogels decreased, which increased the capacitance. Figure 7g showed that the capacitance of the double hydrogel sensor responded to pressure and increased as the pressure increased. Moreover, during instantaneous compression from 1 to 10 kPa, the double hydrogel sensor exhibited good repeatability and a sensitive pressure response.

In addition, to illustrate the signals of the three indicators, 3D colormaps were used for multistimuli sensing and signal distinction. For the lower hydrogel sensor in Figure 7i, the color blocks showed a diagonal trend on all faces of the 3D colormaps, revealing the response to the three stimuli. The 3D color maps of the upper hydrogel sensor only showed a diagonal trend on the face between temperature and blood, whereas the color block changes on the face related to the blood were parallel to the bottom (Figure 7j), which indicated that only temperature and pressure could be monitored through the upper hydrogel sensor. However, the color blocks of the double hydrogel sensors only changed with pressure, indicating a single response to pressure (Figure 7k). Overall, these results demonstrated that this sandwich‐structured hydrogel sensor based on SR‐Gel/ZL enabled real‐time monitoring and signal differentiation of multiple stimulus signals related to the wound temperature, bleeding, and pressure.

## 3 Conclusions

In this study, a multifunctional SR‐Gel system was developed via photopolymerization of a polypseudorotaxane with precisely controlled low host‐guest coverage, SBMA, and NAGA in an aqueous medium. The pulley effect between β‐CD and bile‐acid‐based guest units within the topological network endowed the SR‐Gels with skin‐like mechanical properties, exceptional fatigue resistance, and excellent adaptability to skin deformation. Additionally, the SR‐Gels exhibited repeatable tissue adhesion, effective antibiofouling performance, and good biocompatibility. Furthermore, incorporating levofloxacin‐loaded ZIF‐8 nanoparticles yielded an advanced SR‐Gel/ZL composite, which demonstrated pH‐responsive antibacterial activity and pro‐healing functionality in vivo. A sandwich‐structured hydrogel sensor based on SR‐Gel/ZL was also fabricated, capable of real‐time monitoring and discrimination of wound‐related signals such as temperature variation, bleeding, and pressure changes. This multifunctional sensing capability holds significant potential to improve wound management and therapeutic intervention. Overall, this work overcomes the limitations of conventional SR‐Gels, which often require complex preparation processes, and establishes a new design paradigm for SR materials in smart wound dressing applications.

## 4 Experimental Section

### 4.1 Materials

Lithocholic acid (LCA, 98%), acryloyl chloride (96%), glycinamide hydrochloride (98%), sulfobetaine methacrylate (SBMA, 97%), albumin from bovine serum (BSA, 96%), 2‐hydroxy‐4’‐(2‐hydroxyethoxy)‐2‐methylpropiophenone (I2959, 98%), levofloxacin (98%), 2‐methylimidazole (98%), zinc nitrate hexahydrate (Zn(NO_3_)_2_·6H_2_O, 99%), were purchased from Aladdin (Shanghai, China). Polyethylene glycol (PEG, average M_n_ 2000), ethylenediamine (98%), and β‐cyclodextrin (β‐CD, 98%) were purchased from Macklin (Shanghai, China). VHB elastomer (4910), and Tegaderm film were purchased from 3 M (Shanghai, China). The Micro BCA protein assay kit was purchased from BOSTER Biological Technology Co. Ltd., (Wuhan, China). Cell counting kit‐8, and Calcein AM/PI cell viability/cytotoxicity assay kit were purchased from Beyotime Biotechnology Co. Ltd (Shanghai, China). Anticoagulant rabbit whole blood was purchased from YaJi Biological Co. Ltd. (Shanghai, China).

### 4.2 Synthesis of SR‐Gels

The Synthesis routes of SR‐Gels are shown in the supporting information.

### 4.3 Mechanical Properties Test

Uniaxial tensile tests of SR‐Gels were conducted using a Jinheyuan JHY‐5000 universal tester (China) at room temperature. All samples were cut into rectangular pieces with a dimension of 25 × 10 × 2 mm, and the extension rate was set at 50 mm/min. For the compressive test, the hydrogels were cut into a cylindrical shape (6 mm in diameter and 6 mm in height), and the compression rate was set at 10 mm/min. Stress relaxation tests were conducted on SR‐Gel2 and PEG‐Gel at a pre‐stretched strain of 180% (avoiding PEG‐Gel breakage). Cyclic tensile test of SR‐Gel2 recorded with increased maximum strains from 100% to 700% at a rate of 50 mm/min. One hundred consecutive cyclic loading‐unloading tests were conducted on SR‐Gel2 at a strain of 200% and recorded the continuous change of lord.

### 4.4 Adhesion Properties Test

A SR‐Gel with dimension of 20 × 10 × 2 mm was stuck between two pigskins, and a 100 g weight was placed on substance to tighten the hydrogel for 10 min. The two pigskins were then stretched to failure by a universal tester at a rate of 50 mm/min, and the maximum shear force was recorded. The adhesion strength was calculated by dividing the maximum shear force by the bonding area. In addition, the repeated adhesion properties of the SR‐Gel2 were evaluated through adhesion‐peeling‐adhesion cycle experiments. A burst pressure test was performed using a published method [64]. A 5 mm long incision was made on the surface of the pigskin. Then 100 µL of pre‐gel solution was added to the incision surface, and cured in situ under UV light for 10 min. A syringe pump injected air at a rate of 10 mL/min to recorded the change of pressure. The peak pressure before pressure loss was considered the burst pressure.

### 4.5 Nonspecific Protein Adsorption Measurement

SR‐Gel disks were placed in a 48‐well plate, and 1 mL of BSA solution at a concentration of 2 mg/mL was added. After incubation for 2 h at 37°C, the SR‐Gels were removed from the protein solution. Then, a Micro BCA protein assay kit was used to detect the concentration of remaining BSA in the solution and calculate the protein adsorption of SR‐Gel2.

### 4.6 Drug Release of Hydrogel

SR‐Gel2/ZL hydrogels (10 mm in diameter and 5 mm in height) were soaked in 5 mL of PBS buffer with a pH of 7.4 or 6.0, respectively, at 37°C and 100 rpm. At regular intervals, 1 mL of release solution was collected and replaced it with 1 mL of fresh PBS buffer. The concentration of levofloxacin was obtained by measuring the absorbance at 288 nm directly with a UV‐vis spectrophotometer. The concentration of Zn^2+^ was detected by the zincon spectrophotometric method [65].

### 4.7 Antibacterial Activity of Hydrogel

Antibacterial activity was carried out by the plate‐count method. SR‐Gel2/ZL disks were placed in a 48‐well plate. 100 µL of bacterial suspension (10^6^ CFU/mL) was added to the surfaces of hydrogels. After cultivating for 2 h at 37°C, the disks were washed with PBS buffer and spread out on agar plates to count the number of bacterial colonies. Besides, the antibacterial activity was performed by the inhibition zone method. 100 µL of bacterial suspension (10^6^ CFU/mL) was spread out on agar plates. SR‐Gel2/ZL disks (10 mm in diameter and 5 mm in height) were placed on the surfaced of agar plates. After cultivating for 18 h at 37°C, the diameters of the inhibition zones were measured by vernier calipers.

### 4.8 Hemolysis of Hydrogel

Briefly, hydrogel disks were placed in a 48‐well plate. 1 mL of RBCs suspension at a concentration of 5% v/v was added into each well of hydrogel, and the RBCs suspension mixed with PBS buffer and 0.1% TX‐100 was used as the negative and positive controls, respectively. All of the samples were incubated at 37°C and 100 rpm for 2 h and then centrifuged at 1500 rpm for 5 min. Finally, the OD values at 545 nm of the supernatants were measured using a microplate reader, and the hemolysis ratio was calculated.

### 4.9 Cytotoxicity Assay

The cytotoxicity of hydrogels was carried out with a Cell Counting Kit‐8 (CCK‐8) assay using L929 cells. L929 cells were seeded into a 96‐well plate and incubated for 12 h. Then, 100 µL medium containing the exact was added to each well after removing the medium, and untreated cells incubated with the normal culture medium served as a control. After culturing for 1, 2, and 3 days, the medium was replaced with 100 µL of fresh CCK‐8 kit, followed by a further incubation for 2 h. The OD values at 450 nm were measured using a microplate reader, and the relative cell viability was determined.

### 4.10 In Vivo Wound Healing Assay

According to the method reported by Zhang et al. [66]. An infected full‐thickness skin defect model was used to evaluate the actual ability of the hydrogel to promote infected wound healing. All animal experiments and procedures were performed with the approval of IBMDES23006 and carried out according to the state guidelines from the Ministry of Science and Technology of China. Female SD rats were randomly divided into 3 groups, including Tegaderm film (3 M Health Care, USA) as control, hydrogel SR‐Gel2, and hydrogel SR‐Gel2/ZL_0.3_. 8 mm diameter full‐thickness skin wounds were created by a hole punch. The 20 µL of *S. aureus* suspension was added to the wound sites. After being infected for 24 h, control wounds were dressed with Tegaderm TM film, and hydrogel group wounds were treated with SR‐Gel2 or SR‐Gel2/ZL0.3, and this time was recorded as day 0. After days of treatment, the number of bacteria in wound sites was counted by the plate‐count method using mannitol sodium chloride agar. During the entire experiment, all wounds were photographed at preset time points to record the area of wounds. The wound closure was measured by the ImageJ software and calculated. Besides, histological analysis was performed to further evaluate the wound healing process. All the slices were observed by digital pathology scanner (P250 FLASH, 3D Histech).

### 4.11 Multifunctional Sensing Test of Sensor

The upper/lower layer was SR‐Gel2/ZL_0.3_ (20 × 15 × 2 mm), the middle layer with the same size was a dielectric layer (VHB 4910, 3 M, USA), and electrodes were placed on both sides of the upper/lower hydrogel layer to record the signal changes. A LCR meter (TH2830, Huizhou, China) with a LabView software control was used to detect the resistance of the upper/lower hydrogel layer and the capacitance of the sandwich structure sensor. ΔR/R_0_ (%) and ΔC/C_0_ (%) were calculated to show changes in resistance and capacitance, respectively. Where R_0_ or C_0_ was the initial resistance or capacitance, and ΔR or ΔC was the difference between the real‐time resistance or capacitance and the initial resistance R_0_ or capacitance C_0_.

## Author Contributions

Conceptualization: Wen Huang, Xueru Xiong, Lili Cai, and Yong‐Guang Jia. Methodology: Wen Huang, Xueru Xiong, and Yong‐Guang Jia. Investigation: Wen Huang, Xueru Xiong, and Xiangting Lai. Formal analysis: Wen Huang, Xiangting Lai, Lili Cai, and Lin Wang. Data curation: Wen Huang and Qian Sun. Visualization: Wen Huang, Xiangting Lai, Qian Sun, Yunhua Chen, and Lin Wang. Writing – original draft: Wen Huang and Yong‐Guang Jia. Writing – review & editing: Lili Cai, Lin Wang, and Yong‐Guang Jia. Funding acquisition: Yong‐Guang Jia. Supervision: Lin Wang and Yong‐Guang Jia.

## Conflicts of Interest

The authors declare no conflicts of interest.

## Supporting information




**Supporting File**: advs73635‐sup‐0001‐SuppMat.docx.

## Data Availability

The data that support the findings of this study are available from the corresponding author upon reasonable request.

## References

[1] Y. P. Liang , J. H. He , and B. L. Guo , “Functional Hydrogels as Wound Dressing to Enhance Wound Healing,” ACS Nano 15 (2021): 12687–12722, 10.1021/acsnano.1c04206. 34374515

[2] Z. W. Wang , H. Wei , Y. J. Huang , Y. Wei , and J. Chen , “Naturally Sourced Hydrogels: Emerging Fundamental Materials for next‐generation Healthcare Sensing,” Chemical Society Reviews 52 (2023): 2992–3034, 10.1039/D2CS00813K. 37017633

[3] C. M. Lai , W. J. Chen , Y. Qin , D. Xu , Y. K. Lai , and S. H. He , “Innovative Hydrogel Design: Tailoring Immunomodulation for Optimal Chronic Wound Recovery,” Advanced Science 12 (2025): 2412360, 10.1002/advs.202412360. 39575827 PMC11727140

[4] X. Lei , Y.‐G. Jia , W. Song , et al., “Mechanical and Optical Properties of Reinforced Collagen Membranes for Corneal Regeneration Through Polyrotaxane Cross‐Linking,” ACS Applied Bio Materials 2 (2019): 3861–3869. 10.1021/acsabm.9b00464 35021320

[5] Z. F. Yang , R. K. Huang , B. N. Zheng , et al., “Highly Stretchable, Adhesive, Biocompatible, and Antibacterial Hydrogel Dressings for Wound Healing,” Advanced Science 8 (2021): 2003627. 33898178 10.1002/advs.202003627 PMC8061386

[6] J. Yang , K. Li , C. Tang , et al., “Recent Progress in Double Network Elastomers: One plus One Is Greater than Two,” Advanced Functional Materials 32 (2022): 2110244, 10.1002/adfm.202110244.

[7] L. Y. Long , W. Q. Liu , C. Hu , L. Yang , and Y. B. Wang , “Construction of Multifunctional Wound Dressings With Their Application in Chronic Wound Treatment,” Biomaterials Science 10 (2022): 4058–4076, 10.1039/D2BM00620K. 35758152

[8] Z. Y. Ge , W. S. Guo , Y. Tao , et al., “Wireless and Closed‐Loop Smart Dressing for Exudate Management and On‐Demand Treatment of Chronic Wounds,” Advanced Materials 35 (2023): 2304005, 10.1002/adma.202304005. 37547949

[9] S. A. Eming , P. Martin , and M. Tomic‐Canic , “Wound Repair and Regeneration: Mechanisms, Signaling, and Translation,” Science Translational Medicine 6 (2014): 265sr6, 10.1126/scitranslmed.3009337. 25473038 PMC4973620

[10] D. P. Liu , G. Q. Yin , X. X. Le , and T. Chen , “Supramolecular Topological Hydrogels: from Material Design to Applications,” Polymer Chemistry 13 (2022): 1940–1952.

[11] J. Kim , G. G. Zhang , M. X. Shi , and Z. G. Suo , “Fracture, Fatigue, and Friction of Polymers in Which Entanglements Greatly Outnumber Cross‐links,” Science 374 (2021): 212–216, 10.1126/science.abg6320. 34618571

[12] R. Du , Z. Xu , C. Zhu , et al., “A Highly Stretchable and Self‐Healing Supramolecular Elastomer Based on Sliding Crosslinks and Hydrogen Bonds,” Advanced Functional Materials 30 (2019): 1907139, 10.1002/adfm.201907139.

[13] L. Y. Chen , X. R. Sheng , G. F. Li , and F. H. Huang , “Mechanically Interlocked Polymers Based on Rotaxanes, Chemical Society Reviews,” 51 (2022): 7046–7065. 10.1039/d2cs00202g 35852571

[14] L. F. Hart , J. E. Hertzog , P. M. Rauscher , B. W. Rawe , M. M. Tranquilli , and S. J. Rowan , “Material Properties and Applications of Mechanically Interlocked Polymers,” Nature Reviews Materials 6 (2021): 508–530, 10.1038/s41578-021-00278-z.

[15] X. Liu , J. P. Wu , K. K. Qiao , et al., “Topoarchitected Polymer Networks Expand the Space of Material Properties,” Nature Communications 13 (2022): 1622, 10.1038/s41467-022-29245-0. PMC8956700 35338139

[16] H. Xia , G. Xu , X. Cao , et al., “Single‐Ion‐Conducting Hydrogel Electrolytes Based on Slide‐Ring Pseudo‐Polyrotaxane for Ultralong‐Cycling Flexible Zinc‐Ion Batteries,” Advanced Materials 35 (2023): 2301996. 10.1002/adma.202301996 37339158

[17] D. J. Yoo , A. Elabd , S. Choi , et al., “Highly Elastic Polyrotaxane Binders for Mechanically Stable Lithium Hosts in Lithium‐Metal Batteries,” Advanced Materials 31 (2019): 1901645, 10.1002/adma.201901645. 31148271

[18] A. Bin Imran , K. Esaki , H. Gotoh , et al., “Extremely Stretchable Thermosensitive Hydrogels by Introducing Slide‐ring Polyrotaxane Cross‐linkers and Ionic Groups Into the Polymer Network,” Nature Communications 5 (2014): 5124, 10.1038/ncomms6124. PMC4214411 25296246

[19] K. Iwaso , Y. Takashima , and A. Harada , “Fast Response Dry‐type Artificial Molecular Muscles With [c2]Daisy Chains,” Nature Chemistry 8 (2016): 625–632, 10.1038/nchem.2513. 27219709

[20] H. Xiao , X. T. Lai , X. R. Xiong , et al., “Double‐Network Slide‐Ring Topological Hydrogel Fibers: Fabrication and Sensor Application,” Small 21 (2025): 2501558, 10.1002/smll.202501558. 40095448

[21] L. Jiang , C. Liu , K. Mayumi , K. Kato , H. Yokoyama , and K. Ito , “Highly Stretchable and Instantly Recoverable Slide‐Ring Gels Consisting of Enzymatically Synthesized Polyrotaxane with Low Host Coverage,” Chemistry of Materials 30 (2018): 5013–5019.

[22] C. Liu , N. Morimoto , L. Jiang , et al., “Tough Hydrogels With Rapid Self‐reinforcement,” Science 372 (2021): 1078–1081, 10.1126/science.aaz6694. 34083486

[23] Y. Kobayashi , Y. Nakamitsu , Y. Zheng , Y. Takashima , H. Yamaguchi , and A. Harada , “Control of the Threading Ratio of Cyclic Molecules in Polyrotaxanes Consisting of Poly(ethylene glycol) and α‐cyclodextrins,” Chemical Communications 54 (2018): 7066–7069, 10.1039/C8CC01776J. 29876543

[24] A. Harada , A. Hashidzume , H. Yamaguchi , and Y. Takashima , “Polymeric Rotaxanes,” Chemical Reviews 109 (2009): 5974–6023, 10.1021/cr9000622. 19736930

[25] K. Kato , Y. Okabe , Y. Okazumi , and K. Ito , “A significant impact of host–guest stoichiometry on the extensibility of polyrotaxane gels,” Chemical Communications 51 (2015): 16180–16183, 10.1039/C5CC07122D. 26399995

[26] G. Fleury , C. Brochon , G. Schlatter , G. Bonnet , A. Lapp , and G. Hadziioannou , “Synthesis and Characterization of High Molecular Weight Polyrotaxanes: Towards the Control Over a Wide Range of Threaded α‐cyclodextrins,” Soft Matter 1 (2005): 378–385, 10.1039/b510331b. 32646105

[27] H. Gotoh , C. Liu , A. Bin Imran , et al., “Optically Transparent, High‐toughness Elastomer Using a Polyrotaxane Cross‐linker as a Molecular Pulley,” Science Advances 4 (2018): aat7629, 10.1126/sciadv.aat7629. PMC6184743 30333989

[28] X. R. Xiong , Y. H. Chen , Z. X. Wang , et al., “Polymerizable Rotaxane Hydrogels for Three‐dimensional Printing Fabrication of Wearable Sensors,” Nature Communications 14 (2023): 1331, 10.1038/s41467-023-36920-3. PMC10006079 36898994

[29] T. Zhu , Y. Ni , G. M. Biesold , et al., “Recent advances in conductive hydrogels: Classifications, properties, and applications,” Chemical Society Reviews 52 (2023): 473–509, 10.1039/D2CS00173J. 36484322

[30] J. Y. Cai , M. Zhang , J. Q. Peng , et al., “Peptide‐AIE Nanofibers Functionalized Sutures with Antimicrobial Activity and Subcutaneous Traceability,” Advanced Materials 36 (2024): 2400531. 10.1002/adma.202400531 38716716

[31] K. K. Zheng , Y. Tong , S. H. Zhang , et al., “Flexible Bicolorimetric Polyacrylamide/Chitosan Hydrogels for Smart Real‐Time Monitoring and Promotion of Wound Healing,” Advanced Functional Materials 31 (2021): 2102599, 10.1002/adfm.202102599.

[32] Y. Zhu , J. Zhang , J. Song , et al., “A Multifunctional Pro‐Healing Zwitterionic Hydrogel for Simultaneous Optical Monitoring of pH and Glucose in Diabetic Wound Treatment,” Advanced Functional Materials 30 (2019): 1905493, 10.1002/adfm.201905493.

[33] W. Z. Li , Y. R. Yu , R. K. Huang , et al., “Multi‐Bioinspired Functional Conductive Hydrogel Patches for Wound Healing Management,” Advanced Science 10 (2023): 2301479, 10.1002/advs.202301479. 37376818 PMC10477846

[34] H. S. Guo , M. Bai , Y. N. Zhu , et al., “Pro‐Healing Zwitterionic Skin Sensor Enables Multi‐Indicator Distinction and Continuous Real‐Time Monitoring,” Advanced Functional Materials 31 (2021): 2106406.

[35] J. Liu , H. Y. Wang , T. Liu , et al., “Multimodal Hydrogel‐Based Respiratory Monitoring System for Diagnosing Obstructive Sleep Apnea Syndrome,” Advanced Functional Materials 32 (2022): 2204686, 10.1002/adfm.202204686.

[36] A. Harada , “Construction of supramolecular structures From cyclodextrins, polymers,” Carbohydrate Polymers 34 (1997): 183–188, 10.1016/S0144-8617(97)00023-4.

[37] A. Harada , “Preparation and Structures of Supramolecules Between Cyclodextrins and Polymers,” Coordination Chemistry Reviews 148 (1996): 115–133, 10.1016/0010-8545(95)01157-9.

[38] K. P. Liu , F. J. Zhang , Y. Wei , et al., “Dressing Blood‐Contacting Materials by a Stable Hydrogel Coating With Embedded Antimicrobial Peptides for Robust Antibacterial and Antithrombus Properties,” ACS Applied Materials & Interfaces 13 (2021): 38947–38958, 10.1021/acsami.1c05167. 34433245

[39] Q. S. Liu , A. Chiu , L. H. Wang , et al., “Developing Mechanically Robust, Triazole‐zwitterionic Hydrogels to Mitigate Foreign Body Response (FBR) for Islet Encapsulation,” Biomaterials 230 (2020): 119640, 10.1016/j.biomaterials.2019.119640. 31791840

[40] Y. K. Huang , X. Y. Zhai , T. F. Ma , et al., “A Unified Therapeutic–Prophylactic Tissue‐Engineering Scaffold Demonstrated to Prevent Tumor Recurrence and Overcoming Infection Toward Bone Remodeling,” Advanced Materials 35 (2023): 2300313, 10.1002/adma.202300313. 36939167

[41] W. H. Sun , Z. S. An , and P. Y. Wu , “UCST or LCST? Composition ‐Dependent Thermoresponsive Behavior of Poly,” Macromolecules 50 (2017): 2175–2182.

[42] J. Wu , Z. C. Xiao , A. Q. Chen , et al., “Sulfated Zwitterionic Poly(sulfobetaine methacrylate) Hydrogels Promote Complete Skin Regeneration,” Acta Biomaterialia 71 (2018): 293–305, 10.1016/j.actbio.2018.02.034. 29535009

[43] J. Liu , H. Y. Zheng , P. S. P. Poh , H. G. Machens , and A. F. Schilling , “Hydrogels for Engineering of Perfusable Vascular Networks,” International Journal of Molecular Sciences 16 (2015): 15997–16016, 10.3390/ijms160715997. 26184185 PMC4519935

[44] X. Yang , L. Cheng , Z. M. Zhang , et al., “Amplification of Integrated Microscopic Motions of High‐density [2]Rotaxanes in Mechanically Interlocked Networks,” Nature Communications 13 (2022): 6654, 10.1038/s41467-022-34286-6. PMC9636211 36333320

[45] K. Kato and K. Ito , “Dynamic Transition Between Rubber and Sliding States Attributed to Slidable Cross‐links,” Soft Matter 7 (2011): 8737–8740, 10.1039/c1sm06212c.

[46] K. Mayumi , C. Liu , Y. Yasuda , and K. Ito , “Softness, Elasticity, and Toughness of Polymer Networks with Slide‐Ring Cross‐Links,” gels 7 (2021): 91. 34287305 10.3390/gels7030091 PMC8293080

[47] S. Y. Zheng , C. Liu , L. Jiang , et al., “Slide‐Ring Cross‐Links Mediated Tough Metallosupramolecular Hydrogels With Superior Self‐Recoverability,” Macromolecules 52 (2019): 6748–6755, 10.1021/acs.macromol.9b01281.

[48] R. Song , Z. Liu , X. Geng , L. Ye , A. Zhang , and Z. Feng , “Preparation and Characterization of Cross‐linked Polyurethanes Using β‐CD [3]PR as Slide‐ring Cross‐linker,” Polymer 249 (2022): 124862, 10.1016/j.polymer.2022.124862.

[49] J. Zhao , Z. Zhang , L. Cheng , et al., “Mechanically Interlocked Vitrimers,” Journal of the American Chemical Society 144 (2021): 872–882, 10.1021/jacs.1c10427. 34932330

[50] Z. Y. Yuan , X. C. Duan , X. Su , et al., “Catch Bond‐inspired Hydrogels With Repeatable and Loading Rate‐sensitive Specific Adhesion,” Bioactive Materials 21 (2023): 566–575. 36204280 10.1016/j.bioactmat.2022.09.002 PMC9519436

[51] W. Zhang , B. H. Wu , S. T. Sun , and P. Y. Wu , “Skin‐Like Mechanoresponsive Self‐healing Ionic Elastomer From Supramolecular Zwitterionic Network,” Nature Communications 12 (2021): 4082, 10.1038/s41467-021-24382-4. PMC8253733 34215738

[52] K. Fang , R. Wang , H. Zhang , et al., “Mechano‐Responsive, Tough, and Antibacterial Zwitterionic Hydrogels With Controllable Drug Release for Wound Healing Applications,” ACS Applied Materials & Interfaces 12 (2020): 52307–52318, 10.1021/acsami.0c13009. 33183010

[53] M. B. Perez , D. A. Resendiz‐Lara , Y. Matsushita , et al., “Creating Anti‐Biofouling Surfaces by Degradable Main‐chain Polyphosphoester Polymer Brushes,” Advanced Functional Materials 34 (2024): 2316201, 10.1002/adfm.202316201.

[54] Y. Yu , H. Yuk , G. A. Parada , et al., “Multifunctional “Hydrogel Skins” on Diverse Polymers with Arbitrary Shapes,” Advanced Materials 31 (2019): 1807101. 10.1002/adma.201807101 30570776

[55] L. Mi and S. Y. Jiang , “Integrated Antimicrobial and Nonfouling Zwitterionic Polymers,” Angewandte Chemie International Edition 53 (2014): 1746–1754. 24446141 10.1002/anie.201304060

[56] J. H. Wang , X. Y. Chen , Y. Zhao , et al., “pH‐Switchable Antimicrobial Nanofiber Networks of Hydrogel Eradicate Biofilm and Rescue Stalled Healing in Chronic Wounds,” ACS Nano 13 (2019): 11686–11697. 31490650 10.1021/acsnano.9b05608

[57] M. Yang , W. J. Xu , Z. X. Chen , et al., “Engineering Hibiscus‐Like Riboflavin/ZIF‐8 Microsphere Composites to Enhance Transepithelial Corneal Cross‐Linking,” Advanced Materials 34 (2022): 2109865. 10.1002/adma.202109865 35316534

[58] H. Q. Zheng , Y. N. Zhang , L. F. Liu , et al., “One‐pot Synthesis of Metal–Organic Frameworks With Encapsulated Target Molecules and Their Applications for Controlled Drug Delivery,” Journal of the American Chemical Society 138 (2016): 962–968, 10.1021/jacs.5b11720. 26710234

[59] M. R. Xu , Y. Hu , W. P. Ding , et al., “Rationally Designed Rapamycin‐encapsulated ZIF‐8 Nanosystem for Overcoming Chemotherapy Resistance,” Biomaterials 258 (2020): 120308, 10.1016/j.biomaterials.2020.120308. 32841911

[60] S. Huo , S. Liu , Q. Liu , et al., “Copper–Zinc‐Doped Bilayer Bioactive Glasses Loaded Hydrogel With Spatiotemporal Immunomodulation Supports MRSA‐Infected Wound Healing,” Advanced Science 11 (2023): 2302674, 10.1002/advs.202302674. 38037309 PMC10837387

[61] F. Yang , Y. J. Xue , F. L. Wang , et al., “Sustained Release of Magnesium and Zinc Ions Synergistically Accelerates Wound Healing,” Bioact Mater 26 (2023): 88–101. 36875054 10.1016/j.bioactmat.2023.02.019 PMC9974450

[62] X. M. Liu , S. Tian , S. J. Xu , et al., “A Pressure‐resistant Zwitterionic Skin Sensor for Domestic Real‐time Monitoring and Pro‐healing of Pressure Injury,” Biosensors and Bioelectronics 214 (2022): 114528, 10.1016/j.bios.2022.114528. 35816848

[63] J. S. Mervis and T. J. Phillips , “Pressure Ulcers: Pathophysiology, Epidemiology, Risk Factors, and Presentation,” Journal of the American Academy of Dermatology 81 (2019): 881–890, 10.1016/j.jaad.2018.12.069. 30664905

[64] X. Y. Yin , Y. P. Hao , Y. Lu , et al., “Bio‐Multifunctional Hydrogel Patches for Repairing Full‐Thickness Abdominal Wall Defects,” Advanced Functional Materials 31 (2021): 2105614, 10.1002/adfm.202105614.

[65] S. Yao , J. J. Chi , Y. T. Wang , Y. J. Zhao , Y. Luo , and Y. A. Wang , “Zn‐MOF Encapsulated Antibacterial and Degradable Microneedles Array for Promoting Wound Healing,” Advanced Healthcare Materials 10 (2021): 2100056, 10.1002/adhm.202100056. 33938635

[66] H. Zhang , X. Y. Sun , J. Wang , et al., “Multifunctional Injectable Hydrogel Dressings for Effectively Accelerating Wound Healing: Enhancing Biomineralization Strategy,” Advanced Functional Materials 31 (2021): 2100093.

