# Supplementary material for: Construction of Skin‐Adaptable Slide‐Ring Hydrogels Based on Bile Acids Derived Polyrotaxanes for Smart Wound Dressing
Source: Adv Sci (Weinh). 2025 Dec 27;13(14):e20750. doi: 10.1002/advs.202520750 (PMC12970262; doi:10.1002/advs.202520750)
Supplement: Supplementary file 1 — Supporting File: advs73635‐sup‐0001‐SuppMat.docx. [file ADVS-13-e20750-s001.docx]

Supplementary Information

**Construction of Skin-Adaptable Slide-Ring Hydrogels Based on Bile Acids Derived Polyrotaxanes for Smart Wound Dressing**

*Wen Huang ^1,3^, Xueru Xiong ^2,3^, Qian Sun ^2,3^, Xiangting Lai ^2^, Lili Cai^4*^, Yunhua Chen^2,3^, Lin Wang ^2,3*^, Yong-Guang Jia^5*^*

^1^School of Biomedical Science and Engineering, South China University of Technology, Guangzhou 510006, China

^2^School of Material Science and Engineering, South China University of Technology, Guangzhou 510006, China

^3^National Engineering Research Center for Tissue Restoration and Reconstruction, South China University of Technology, Guangzhou 510006, China

^4^School of Life Science, Zhuhai College of Science and Technology, Zhuhai 519040, China

^5^Center for Advanced Materials Research, Beijing Normal University, Zhuhai 519087, China

**Correspondence:** Lili Cai (lilicai2046@126.com), Lin Wang ([wanglin3@scut.edu.cn](mailto:wanglin3@scut.edu.cn)), Yong-Guang Jia (ygjia@bnu.edu.cn)

**Experimental Section**

The Synthesis routes of polymerizable polypseudorotaxane crosslinker (PPR) was shown in Figure S1.


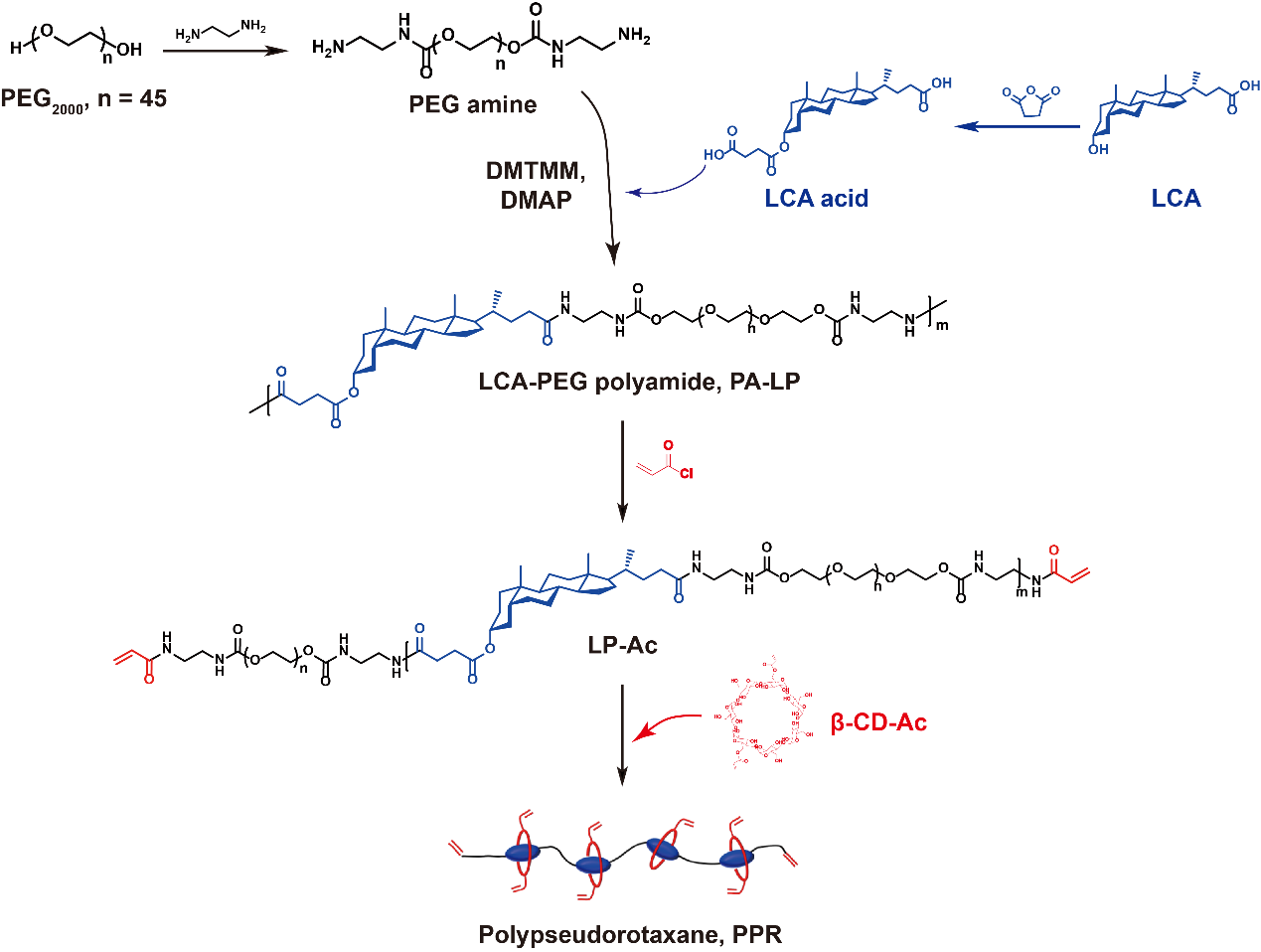


**Figure S1.** Synthesis routes of polymerizable polypseudorotaxane crosslinker (PPR).

*Synthesis of PEG amine:* PEG (6 g, 3 mmol) and CDI (1.216 g, 7.5 mmol) were dissolved in 25 mL of DCM, and stirred at room temperature for 3 h. Then, Ethylenediamine (1.803 g, 2 mL) was added dropwise to the above solution and refluxed at 50 ℃ for 12h. The mixture was precipitated, washed with ethyl ether, and then dried under vacuum overnight to obtained a white powder product noted as PEG amine (Yield: 84%).

*Synthesis of LCA-acid:* LCA (5 g, 13.3 mmol), succinic anhydride (3.99 g, 39.9 mmol) and triethylamine (2.03 g, 20 mmol) was added into 50 ml of chloroform, sonicated until dissolved, and refluxed at 60 ℃ for 24 h. The chloroform was removed by rotavapor and 30 mL of DMSO was added to re-dissolve the crude product. Next, the above solution was added dropwise to deionized water of pH 3.0, and a white precipitate immediately appeared. This precipitate was filtered, washed with deionized water, and then dried under vacuum to obtain LCA-acid (Yield: 86%).

*Synthesis of LCA-PEG Polyamide:* LCA-COOH_2_ (119.14 mg, 0.25 mmol), PEG amine (597.36 mg, 0.275 mmol), DMTMM (103.77 mg, 0.375 mmol), and DMAP (6.11 mg, 0.05 mmol) were dissolved in 4 mL of DMSO and stirred for 12 h. Next, the above solution was stirred for 24 h at 50 ℃. After the reaction, the mixture was dialyzed exhaustively (MWCO 2000) against deionized water for 3 days. The white product named LCA-PEG polyamide (LP) was obtained by lyophilization (Yield: 43%).

*Synthesis of LP-Ac:* LCA-PEG polyamide (1.0 g, 0.06 mmol), triethylamine (60.71 mg, 0.6 mmol) was dissolved in the 5 mL DCM and stirred for 30 min at nitrogen atmosphere and ice bath. Then, acryloyl chloride (54.3 mg, 0.6 mmol) was added dropwise. After stirred for 24 h, the mixture was dialyzed exhaustively (MWCO 2000) against deionized water for 3 days. The pale-yellow product named LP-Ac was obtained by lyophilization (Yield: 54%).

*Synthesis of β-CD-Ac:* β-CD (4.53 g, 4 mmol) and triethylamine (890.47 mg, 8.8 mmol) was dissolved in the 30 mL DMF and stirred for 30 min at nitrogen atmosphere and ice bath. Then, acryloyl chloride (796.4 mg, 8.8 mmol) was added dropwise. After reaction for 12 h, the mixture was filtered to remove the precipitate. The filtrate was precipitated, washed with acetone, and then dried under vacuum overnight to obtained a white product noted as β-CD-Ac (Yield: 77%).

*Synthesis of PPR:* LP-Ac (600 mg, 0.228 mmol) and β-CD-Ac (283.4 mg, 0.228 mmol) was dissolved in 20 mL of deionized water and stirred overnight. Then, the light-yellow product named PPR was obtained by lyophilization (Yield: 95%).

*Synthesis of NAGA:* NAGA was prepared as the reference^[1]^. Briefly, in a 500 mL three-necked flask, glycinamide hydrochloride (18.9 g, 171 mmol) was dissolved in 120 mL of 2 M K_2_CO_3_ solution. Then, 60 mL ethyl ether was added with fast stirring at nitrogen atmosphere and ice bath. Next, acryloyl chloride (18.57 g, 205.2 mmol) was diluted with 30 ml ethyl ether and added dropwise. After reaction for 4 h, the mixture was adjusted to pH 2.0 with 6 M HCl and extracted three times with ethyl ether to remove the organic phase. The aqueous phase was then adjusted to pH 7.0 with 2 M NaOH solution. Subsequently, the white crude product was obtained by lyophilization. 600 mL solvent of methanol : ethanol with a volume ratio of 1 : 4 was used to disperse the lyophilized product. The precipitate was removed by filtration, and the filtrate was concentrated by rotavapor. Then the mixture was recrystallized at 4 ℃ and dried under vacuum overnight to obtain a white powder named NAGA (Yield: 79%).

*Synthesis of ZIF-8@Levo Nanoparticles:* The ZIF-8@Levo was prepared as the reference^[2]^. Briefly, 2-methylimidazole (6.15 g, 0.075 mmol) was dissolved into 45 mL of deionized water. Zn(NO_3_)·6H_2_O (0.372 g, 1.25 mmol) and levofloxacin (150 mg) was dissolved into another 5 mL of deionized water and added to the above solution. After intensely stirred for 1 hour at room temperature, the suspension was centrifuged and washed with methanol three times. The products were then dried under vacuum overnight (Yield: 86%).

*Synthesis of SR-Gels:* An appropriate mass of SBMA, NAGA and PPR crosslinker was first dissolved in deionized water according to the specified recipes (Table 1). Then 1 wt% I2959 was added into the solutions and stirred thoroughly to completely dissolved. After that the mixtures were added into the Polytetrafluoroethylene (PTFE) moles. The polymerization was carried out with the UV light (365 nm, 45 W) induced for 10 min. A series of SR-Gels were obtained by varying feed ratios. It was noted that the reactant content here was mass-to-volume ratio. As for SR-Gel/ZL hydrogels, the ZIF-8@Levo was dispersed into deionized and added into the pre-gel solution before polymerization.

*Characterizations.* ^1^H and 2D NOESY NMR spectra were recorded on a Bruker AVANCE III HD 400 NMR spectrometer at room temperature. FTIR spectra were recorded on a Thermo-Fisher iS50 ATR-FTIR spectrometer in the spectral range of 4000-400 cm^-1^. The hydrogel samples were soaked into deionized water for three days to remove unreacted monomers and lyophilized in a freeze dryer for test. UV-vis spectra were recorded on a Shimadzu UV 2600. The phase of hydroxyapatite was

determined using an Empyrean XRD equipped with Cu Kα radiation (λ = 1.54178 Å). XRD data were recorded from 3-40° for PPR and 5-60° for nanoparticles and hydrogels (2θ) with a scan rate of 5°/min. The morphology of nanoparticles was observed using a Zeiss Merlin FE-SEM. Thermogravimetric analysis (TGA) and differential scanning calorimetry (DSC) measurements were performed on a Netzsch STA 449 F5 under nitrogen atmosphere from 25-700 ℃ at a heating rate of 10K/min. The OD values were measured by a Thermo-Fisher Varioskan Flash 3001 microplate reader. The swelling properties of hydrogels were measured at 37 ℃ using the reported gravimetric method^[3]^. The water contact angle of hydrogels was measured by a contact angle goniometer (OCA15, Data Physics).

*Rheological Properties Test:* Anton Paar MCR302 was used to determine the rheological properties of SR-Gel2 and PEG-Gel. For the frequency sweep tests, the constant strain was set as 1% and the frequency increased logarithmically from 0.1 to 100 rad/s. For the strain sweep tests, the frequency was set as 10 rad/s and the constant strain increased logarithmically from 0.1 to 1000%. For the temperature sweep tests, the frequency and constant strain were set as 10 rad/s and 1%, respectively, and the temperature increased linearly from 25 to 80 ℃.

*Water Angle Test:* The water angle of SR-Gels and PEG-Gel was measured by a contact angle goniometer (OCA15, Data Physics). Briefly, the surface of prepared hydrogel was purged with N_2_ first. Then about 3.0 μL of water droplets was put on the hydrogel in the air, and the contact angle was measured between the water droplets and the hydrogel.

*In Vitro Cell Adhesion Evaluation:* The cell adhesion on the surface of SR-Gels was tested by using L929 cells. The SR-Gel disks were placed in a 48-well plate, and L929 cells were seeded into each SR-Gel2 disk qt a density of 1×10^4^ cells/well. After incubated for 24 h at 37 ℃ in a 5% CO_2_ atmosphere, the SR-Gels were gently rinsed with PBS buffer to remove the suspended cells. Then, the Calcein-AM staining kit was added into the SR-Gels disk to incubated for 15 min at 37 ℃. The morphology of attached L929 cells were observed by an inverted fluorescence microscope (Axio Observer A1, Zeiss).

*In Vitro Antifouling Ability Against Whole Blood:* Antifouling ability against whole blood was performed using a published method^[4]^. First, the commercially available traditional gauze dressings were soaked in the pre-gel solution of SR-Gel2 for 5 min, and then cured under UV light for 10 min to prepare SR-Gel2 coated dressings. Next, the SR-Gel2 coated dressings were bathed in rabbit whole blood for 2 h at 37 ℃。After that, the dressings were then removed and washed gently with PBS buffer three times to remove unabsorbed blood. The dressings without SR-Gel2 coating were used as comparisons.

Drug Loading estimation: To determine the DLE and EE, the dried ZIF-8@Levo with appropriate quantity was decomposed completely in 200 μL of HCl (1.0 mol/L) and then diluted into used solvent. The resulted sample was then evaluated through UV-Vis spectrophotometry at wavelength of 288 nm using standard calibration curve. The DLE and EE of ZIF-8@Levo were calculated using the formulae given below.

Drug Loading Efficiency (DLE, %) = (quantity of levofloxacin loaded) / (quantity of ZIF-8@Levo NPs) × 100%

Entrapment Efficiency (EE, %) = (quantity of levofloxacin loaded) / (total quantity of feeding drug) × 100%

*Bacteria Antifouling Ability Test:* The bacteria antifouling ability on the surface of SR-Gels was tested by using E. coli and S. aureus. The SR-Gel disks were placed in a 48-well plate. 1 mL of bacterial suspension (10^8^ CFU/mL) of *E. coli* or *S. aureus* was added to each well and incubated for 6 h. Then, the SR-Gel disks were clamped out and gently washed with PBS buffer three times to remove unattached bacteria. The adhered bacteria on the surface of SR-Gel were treated with FDA staining kit. The amounts of attached bacteria were observed by an inverted fluorescence microscope and quantified by Image J software.

**Supporting Figures**


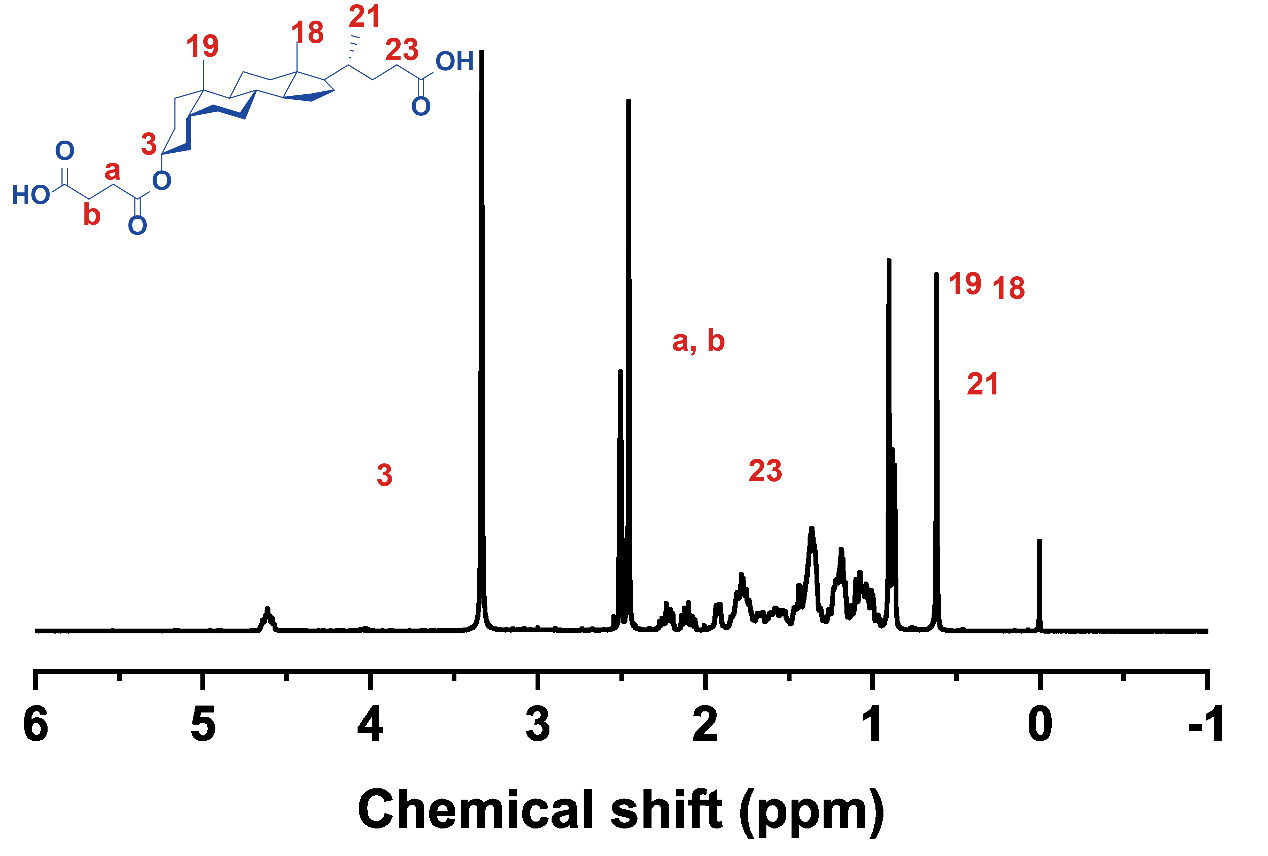


**Figure S2.** ^1^H NMR spectrum of LCA acid in DMSO-d6 as well as the assignments of peaks.


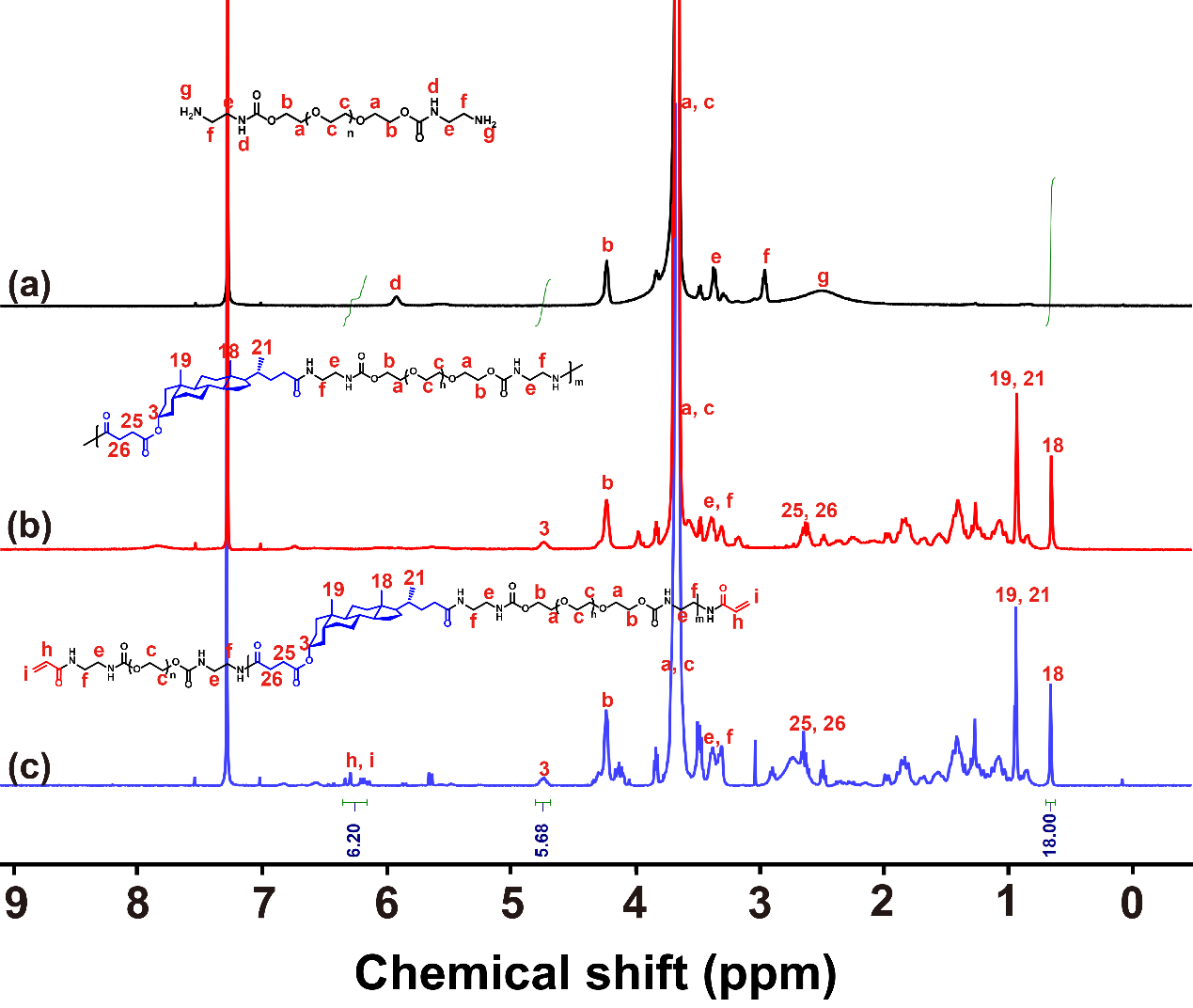


**Figure S3.** ^1^H NMR spectra of (a) PEG amine, (b) PA-LP and (c) LP-Ac in CDCl_3_ and the assignments of peaks. The numbers of LCA units on each linear polymer chain for LP-Ac were estimated to be ca. 6 based on the integration ratio of peaks h and i to peak 18.


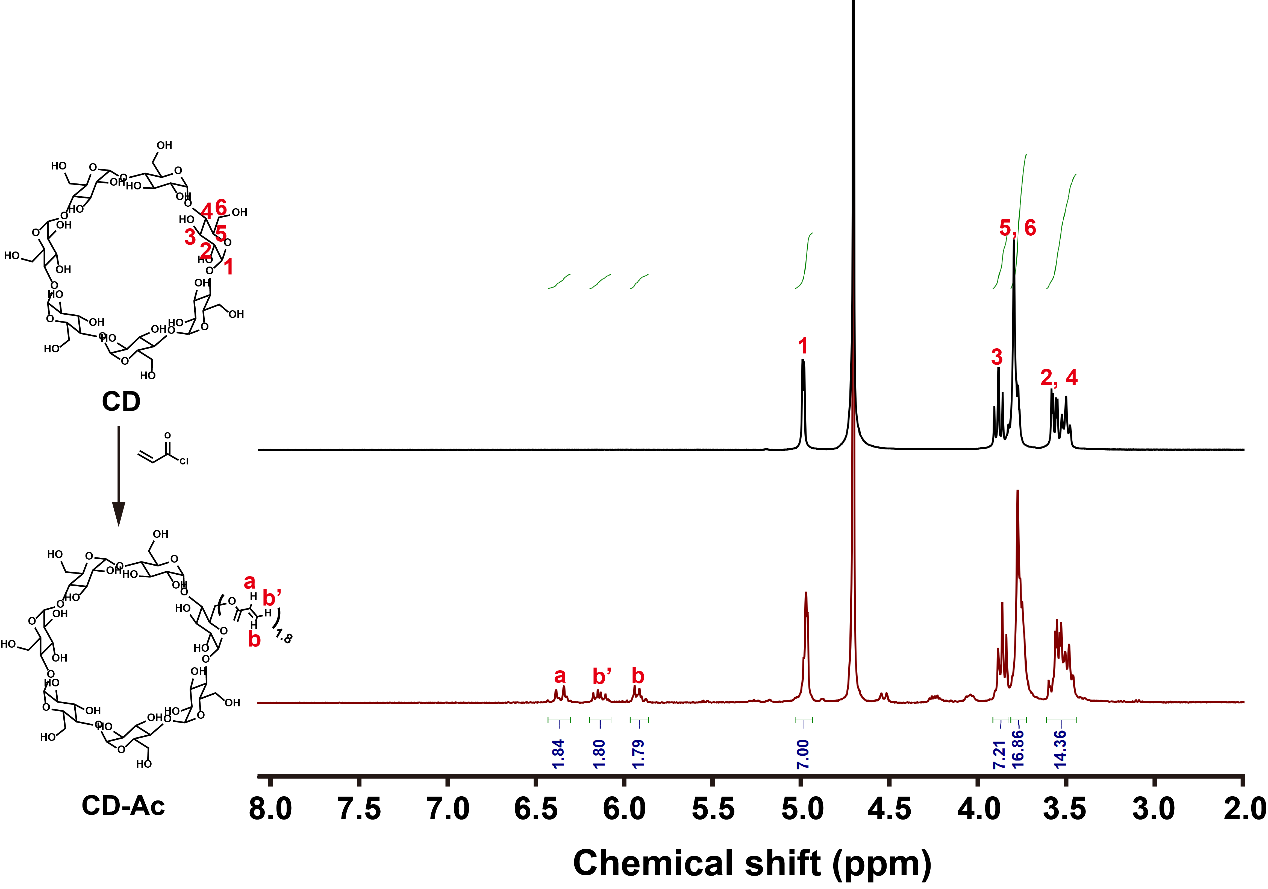


**Figure S4.** Synthesis route of CD-Ac and its ^1^H NMR spectrum obtained in D_2_O as well as the assignments of peaks. Acrylate units on each CD-Ac were estimated to be ca. 1.80 based on the integration ratio of peaks a, b or b’ to peak 1.


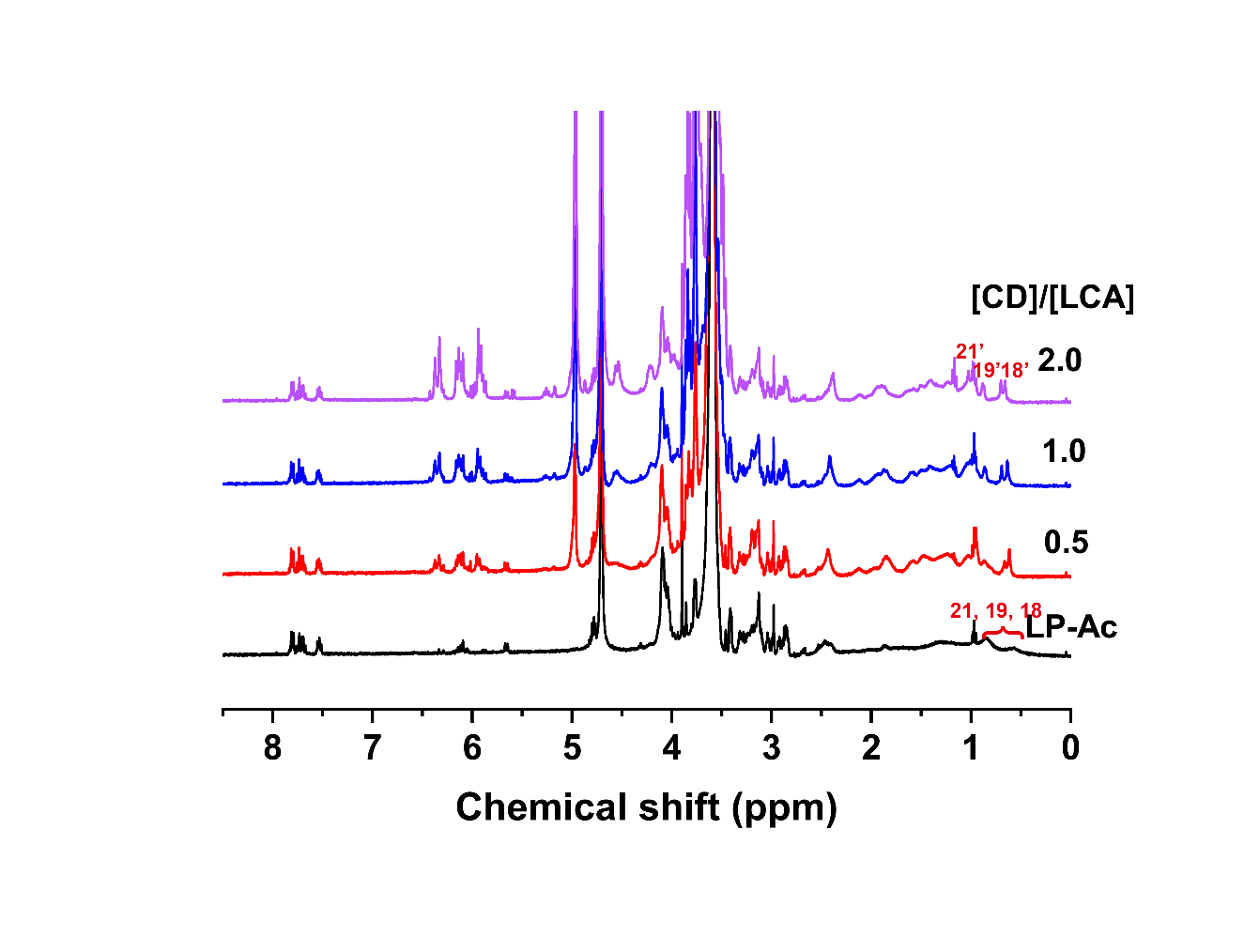


**Figure S5.** ^1^H NMR spectra of LP-Ac in the presence of CD-Ac in D_2_O. The signals of the three methyl protons on the CA moieties all shift downfield and become sharper, indicating the formation of host-guest complexes.


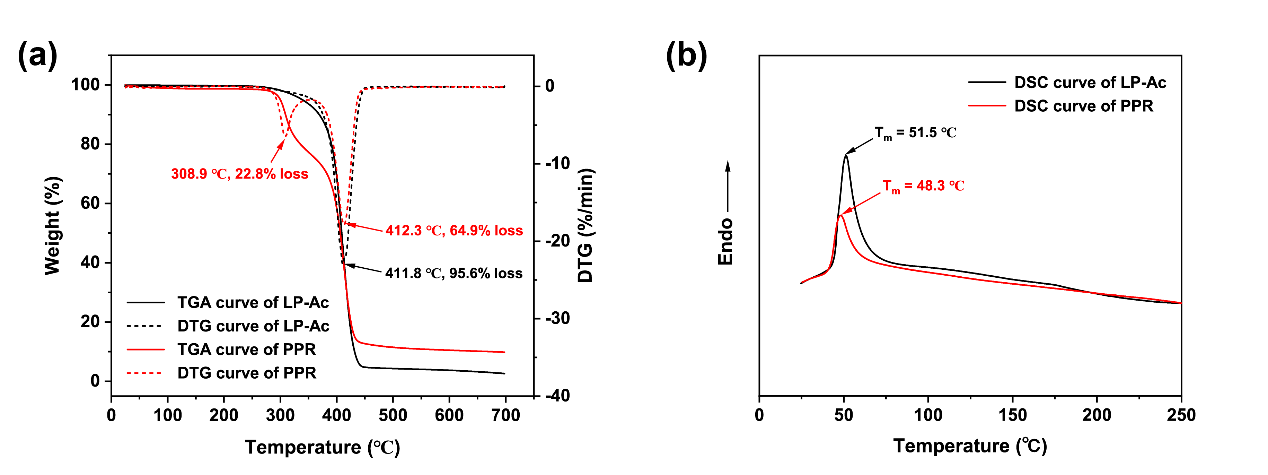


**Figure S6.** (a) TG and DTG curves of LP-Ac and its PPR toward CD-Ac (heating run at 10 K/min under nitrogen atmosphere). Lp-Ac underwent one-step thermal degradation, while its PPR underwent two-step thermal degradation. The first step degradation in the PPR was mainly attributed to decomposition of CD-Ac. (b) DSC curves of LP-Ac and its PPR toward CD-Ac (heating run at 10 K/min under nitrogen atmosphere). Both of them present T_m_ for the PEG blocks at 51.5 and 48.3 ℃, respectively. Above results indicated that the PEG blocks in PPR still remained crystalline, but the complexation of CD-Ac reduced the crystallinity of PEG, resulting in a lower T_m_, which was consistent with XRD results shown in Figure 1b.


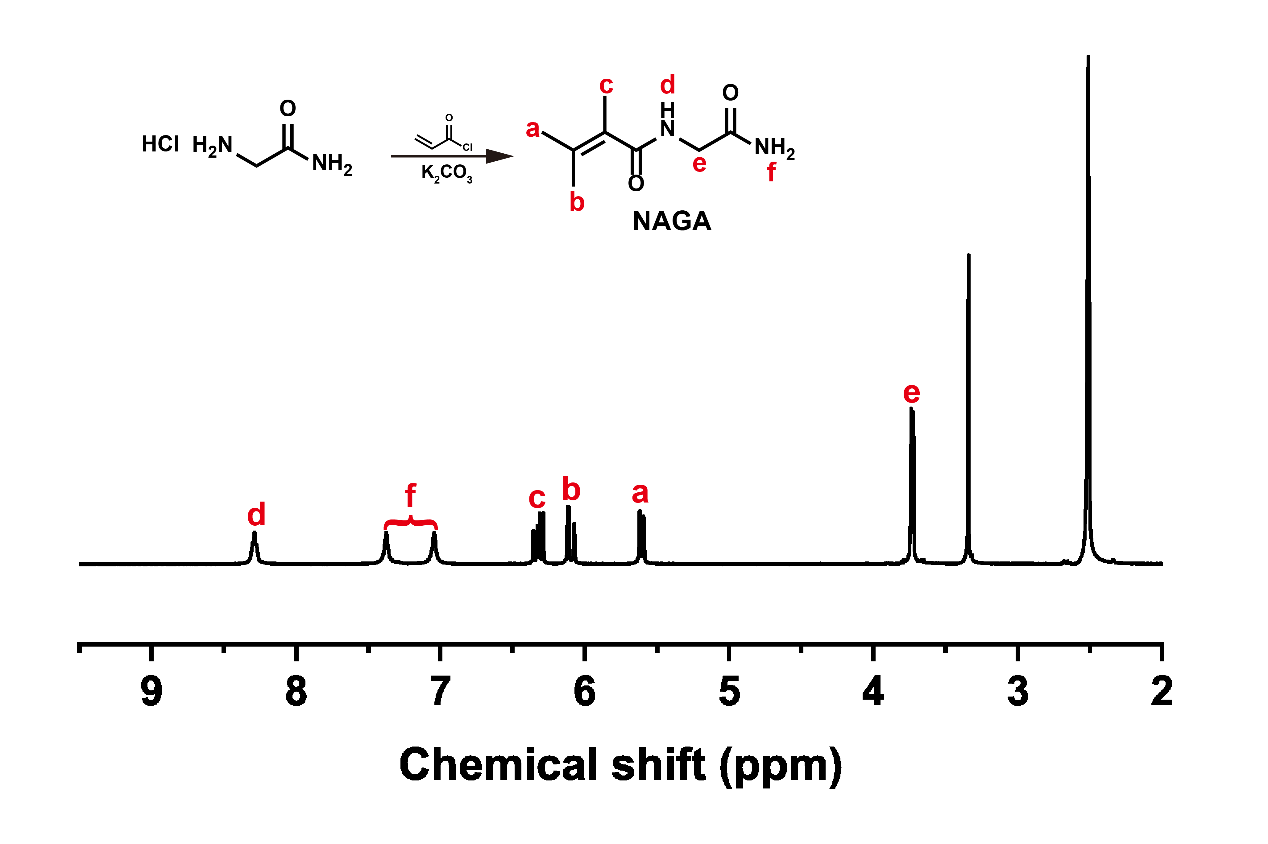


**Figure S7.** Synthesis route of NAGA and its ^1^H NMR spectrum in DMSO-d6 as well as the assignments of peaks.


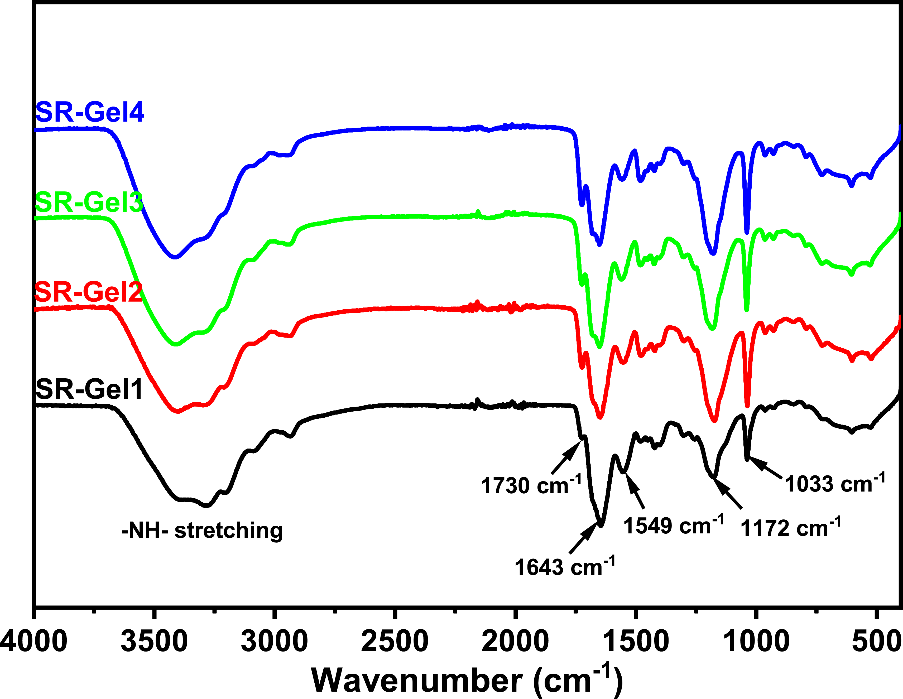


**Figure S8.** FTIR spectra of SR-Gel with different mass composition.


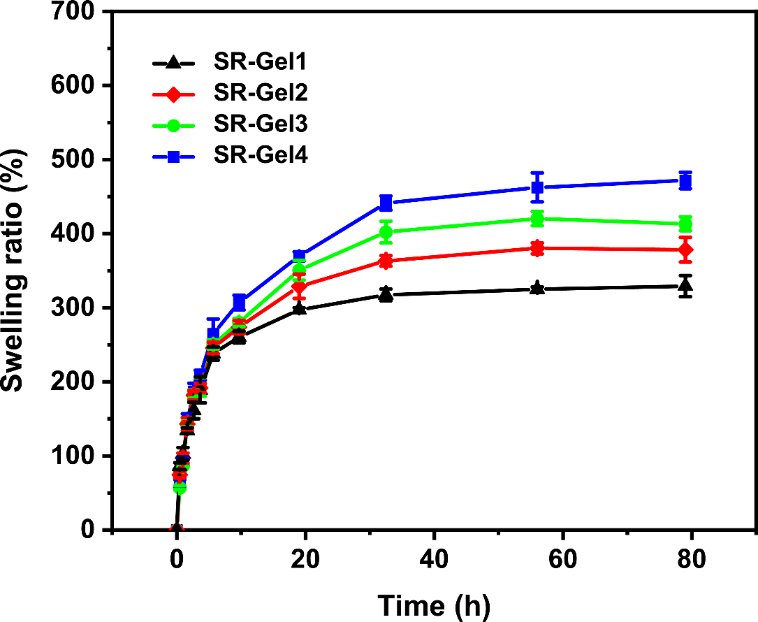


**Figure S9.** Swelling curves of SR-Gel (fixed the total monomer and PPR concentration at 60 w/v% and 0.6 w/v%, respectively). Values are expressed as the means ± SD (n = 3).


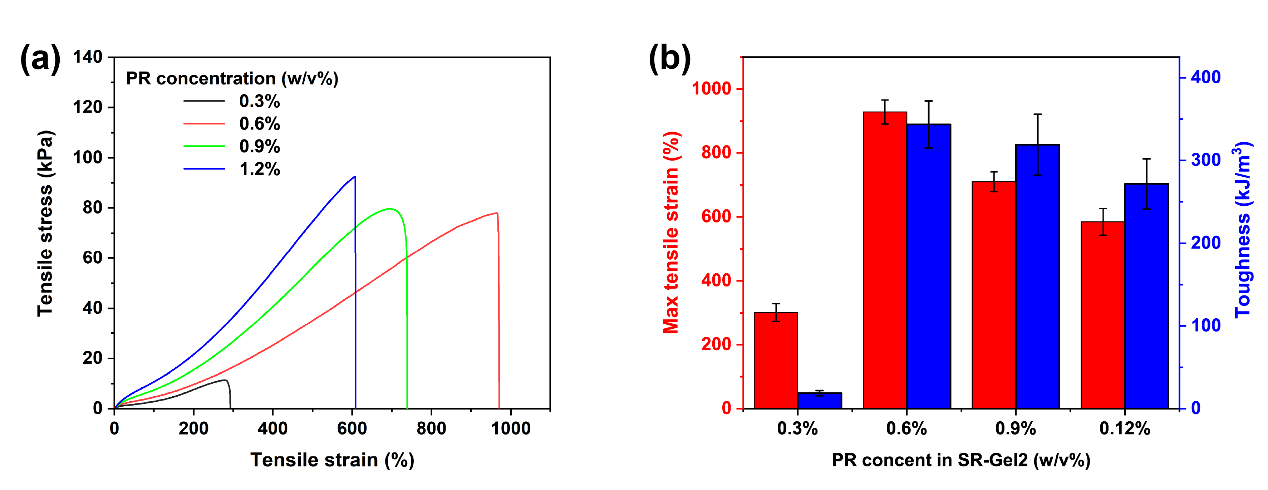


**Figure S10.** (a) Tensile stress-strain curves and (b) its max tensile strain and toughness of SR-Gel with different PR concentrations (fixed the SBMA and NAGA concentration at 30 w/v%, respectively), indicating the SR-Gel with 0.6 w/v% PR network had the best mechanical properties. Values are expressed as the means ± SD (n = 3).


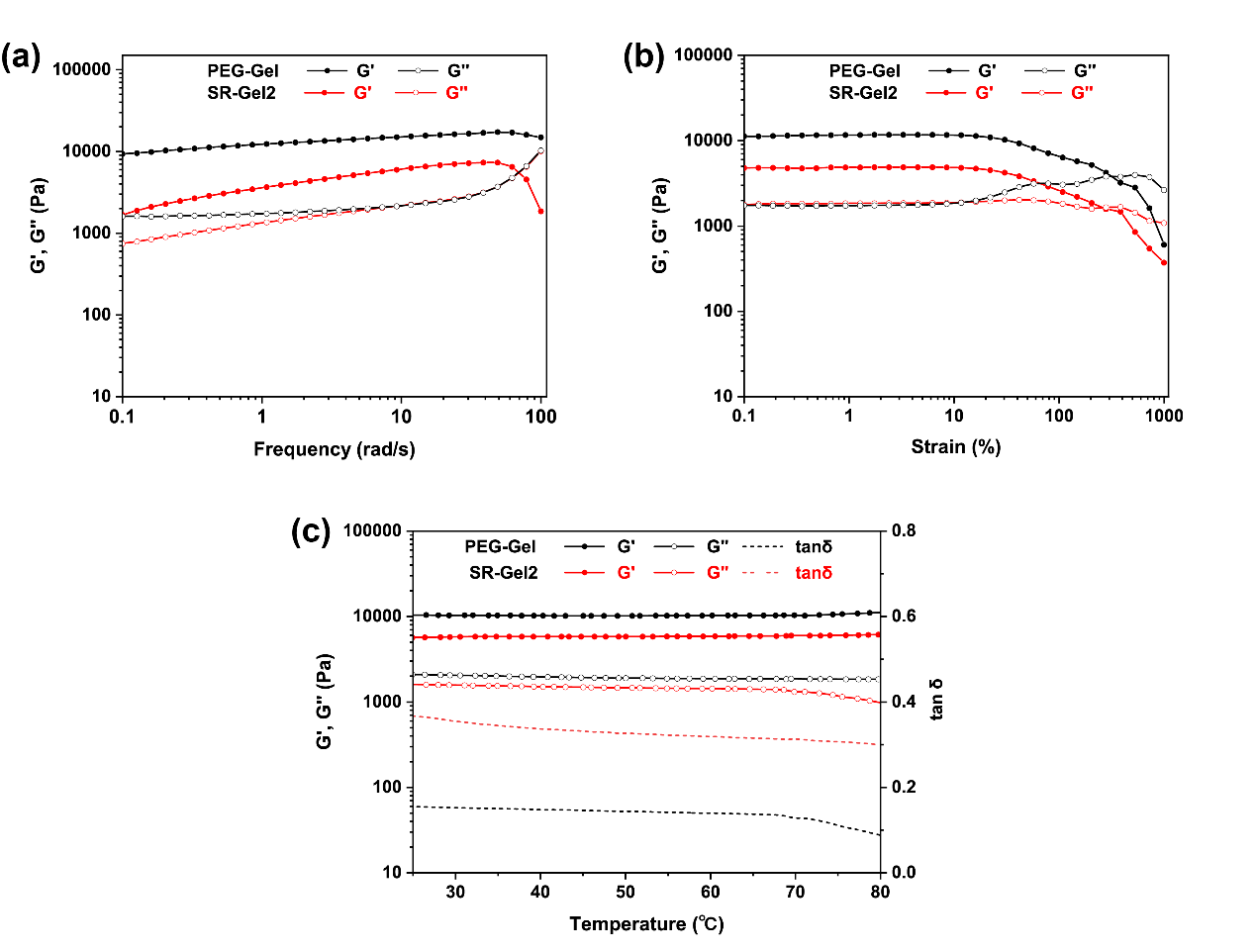


**Figure S11.** Rheological properties of SR-Gel2 and PEG-Gel: (a) frequency sweep, (b) strain sweep and (c) temperature sweep. SR-Gel2 occurred gel-sol transition at lower frequency (68.5 vs more than 100 rad/s) and strain (244.6 vs 327.1%) than that of PEG-Gel, indicating the higher fluidity of SR-Gel network. The tan δ value of SR-Gel was obviously larger than PEG-Gel, meaning that greater energy dissipation existed in SR-Gel network.


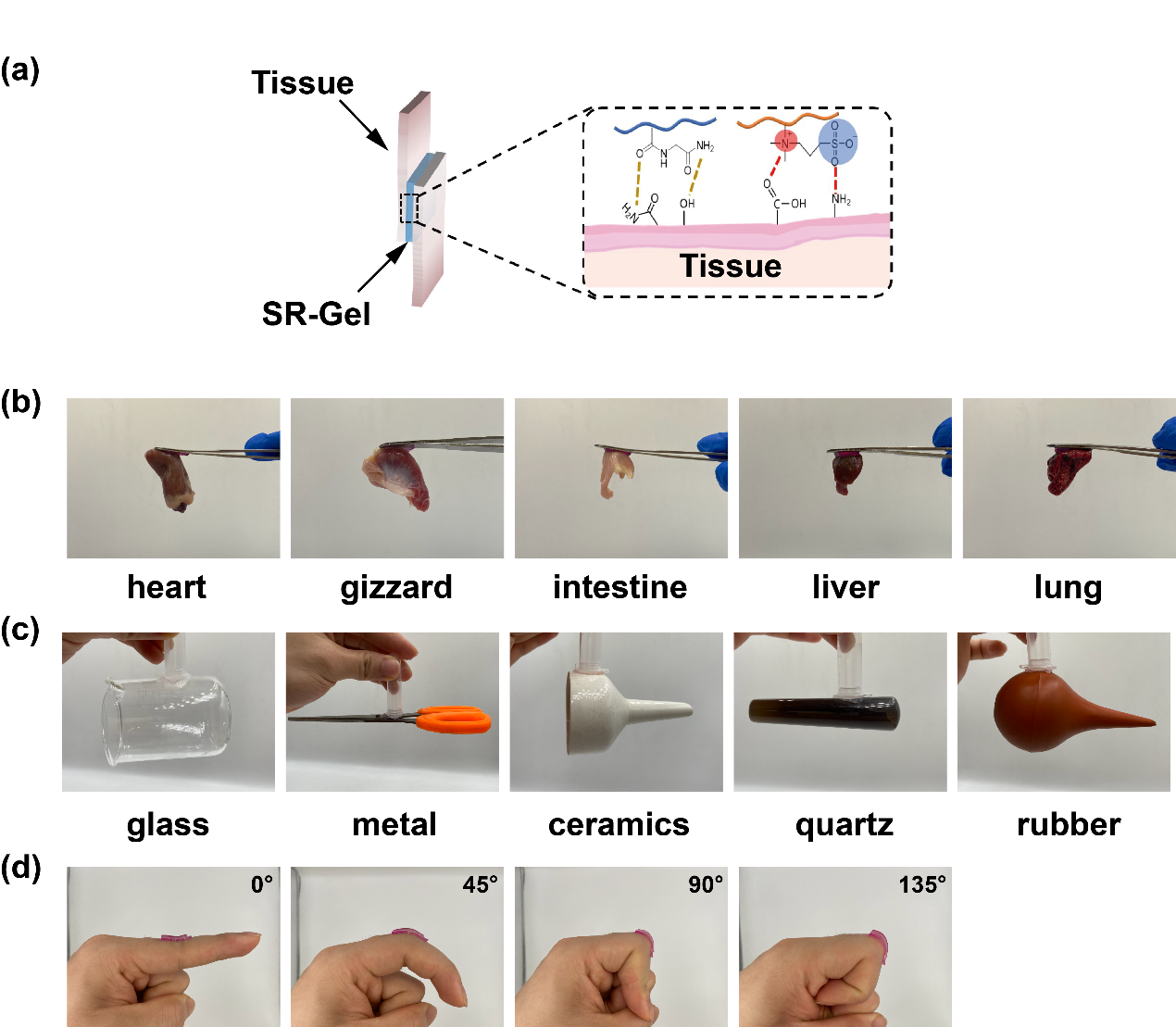


**Figure S12.** (a) Schematic diagram of the adhesion mechanism of SR-Gel. (b) Adhesion of SR-Gel to different tissues. (c) Adhesion of SR-Gel to different substances. (d) The adhesion of SR-Gel on skin under different degrees of finger deformation (from 0° to 45°, 90° and 135°).


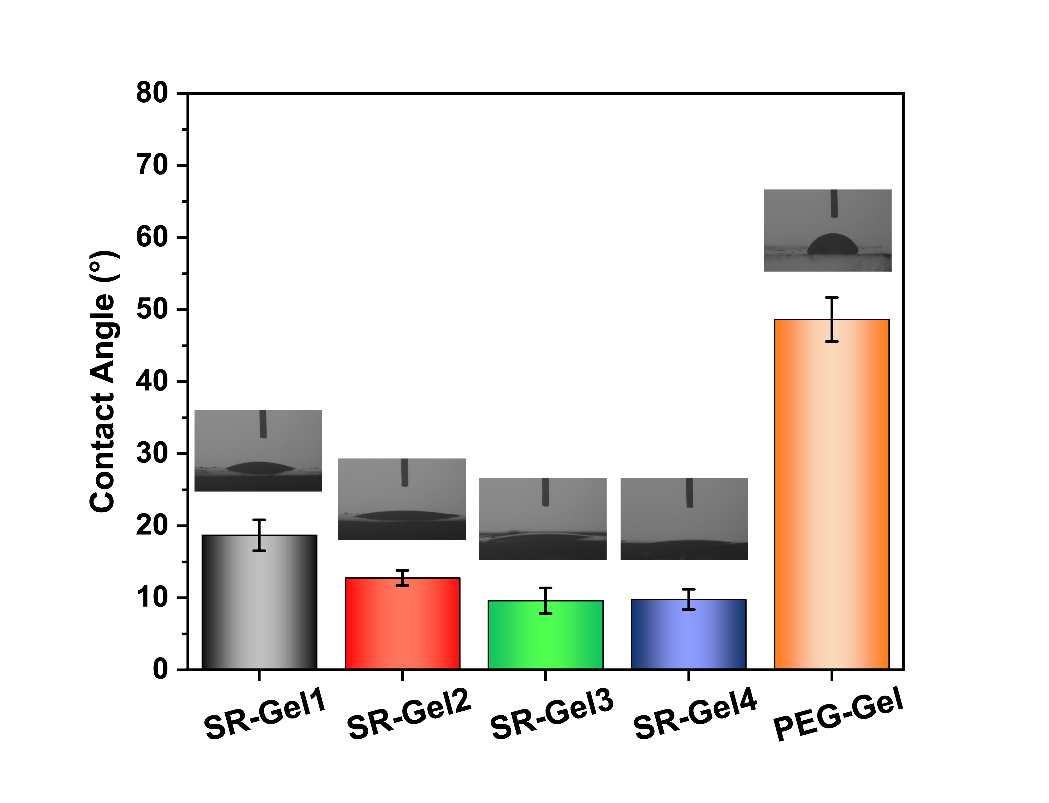


**Figure S13**. The contact angle of SR-Gel and PEG-Gel. As the SBMA content increased, the hydrophilicity of the SR-Gel gradually increased. Values are expressed as the means ± SD (n = 3).


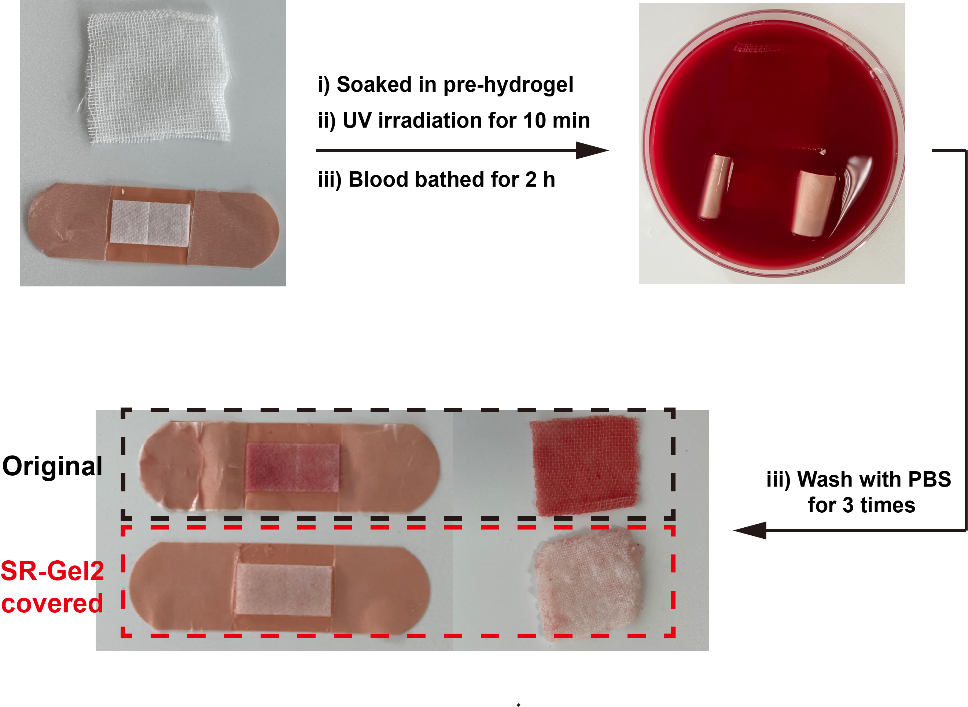


**Figure S14.** Photographs of whole blood cell attachment on the surface of dressings. Traditional dressings and SR-Gel2 coating dressings were soaking in whole blood for 2 h and washed after incubation. Results showed that traditional dressings were heavily contaminated by whole blood, while SR-Gel2 coatings were able to resist most blood contaminations.


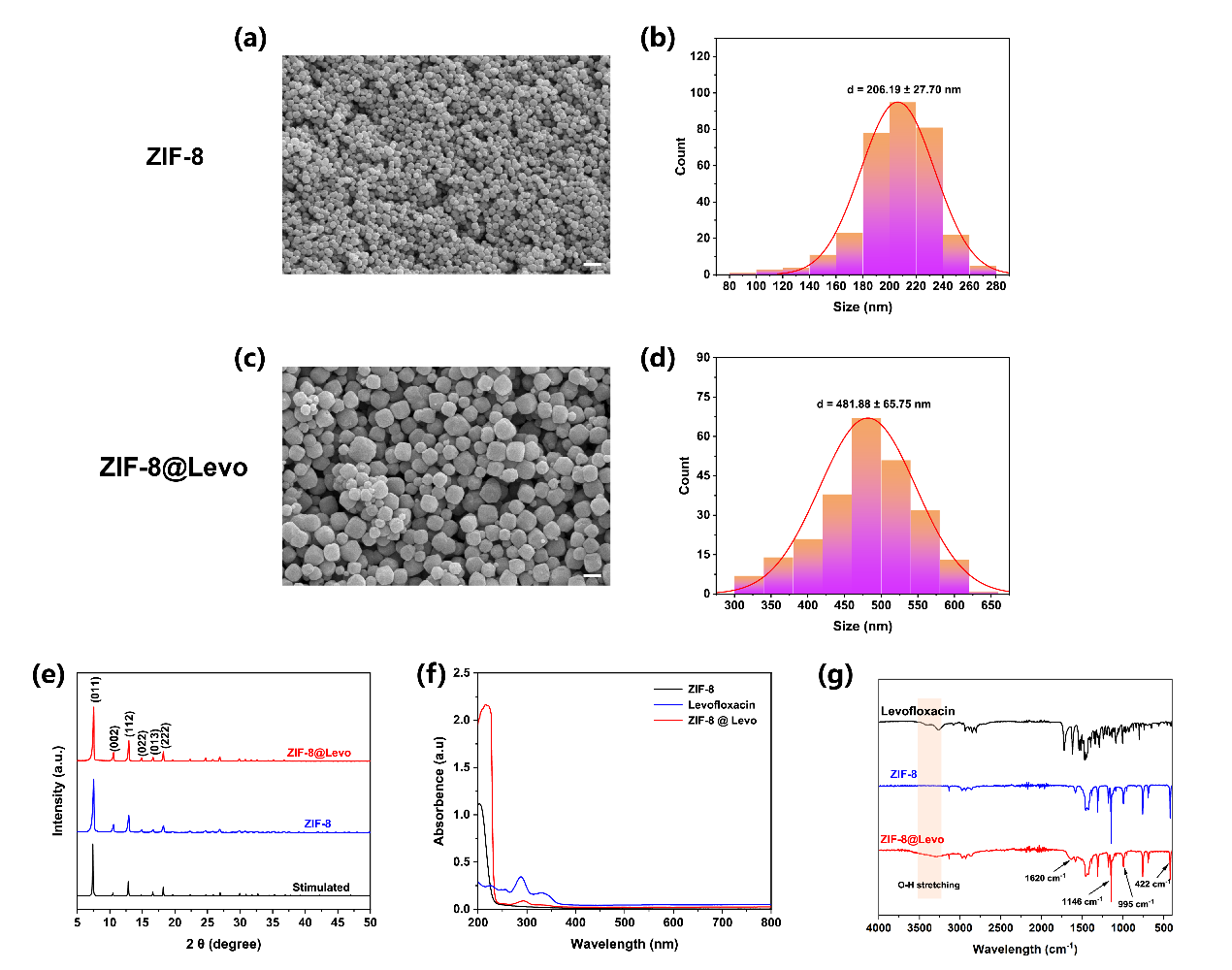


**Figure S15.** (a) SEM image and (b) particle size distribution of ZIF-8 (the scale bar was 500 nm). (c) SEM image and (d) particle size distribution of ZIF-8@Levo, it showed a large diameter after drug loaded (scale bar, 500 nm). (e) Powder XRD patterns of ZIF-8 and ZIF-8@Levo were similar to the published simulation patterns of ZIF-8 crystal structure^[5]^. (f) The UV-vis spectra of levofloxacin, ZIF-8 and ZIF-8@Levo, the absorption peak (around 218 nm) was related to zinc element of ZIF-8, the absorption peak (around 288 nm) was derived from levofloxacin^[6]^. (g) The FT-IR spectra of levofloxacin, ZIF-8 and ZIF-8@Levo, the peaks at 995 cm^-1^ and 1146 cm^-1^ were attributed to the stretching vibrations of the C-N bonds of the imidazole groups, and the characteristic peak at 442 cm^-1^ was attributed to the formation of the Zn-N coordination bonds between Zn^2+^ and the imidazole groups. In addition, characteristic peaks at 1620 cm^-1^ and 3200-3500 cm^-1^ appeared in ZIF-8@Levo, which were attributed to the stretching vibrations of the C=O bonds and O-H bonds in levofloxacin, respectively^[7]^. These results indicated that levofloxacin was successfully loaded into ZIF-8, and drug loading did not disrupt the crystal structure of ZIF-8.


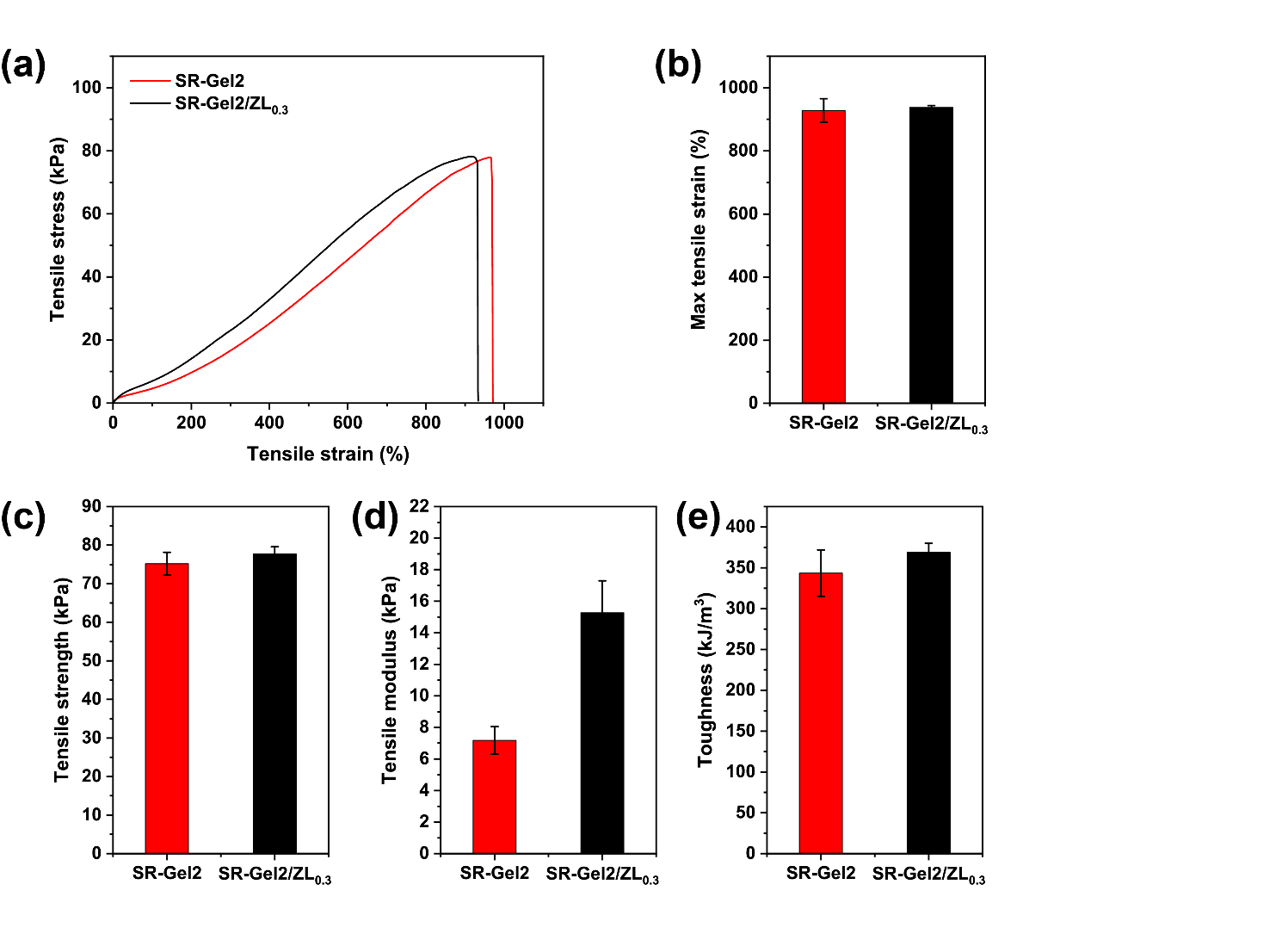


**Figure S16.** (a) Tensile stress-strain curves, (b) max tensile strain, (c) tensile strength, (d) tensile modulus and (e) toughness of SR-Gel2 and SR-Gel2/ZL0.3. Values are expressed as the means ± SD (n = 3).


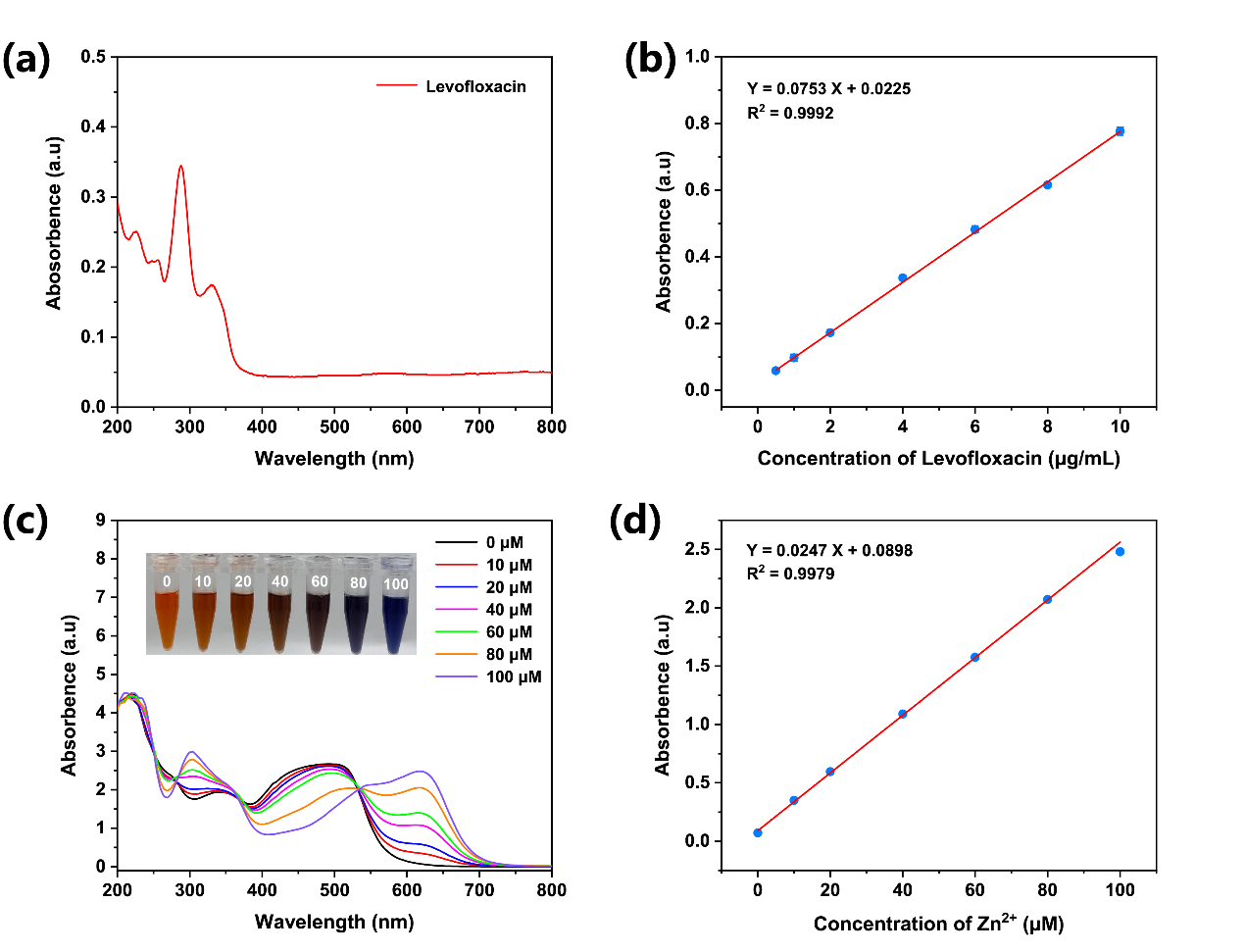


**Figure S17.** The levofloxacin release test was performed by the UV-vis method. (a) The UV-vis spectrum of levofloxacin, and the maximum absorption peak was located at 288 nm. (b) Standard curve of levofloxacin at 288 nm. The Zn^2+^ release test was performed by the zincon spectrophotometric method, Zn^2+^ and zincon would react to produce a blue complex. (c) The UV-vis spectra of Zn^2+^ - zincon complex, and a new absorption peak appeared at 620 nm. (d) Standard curve of Zn^2+^ at 620 nm. Values are expressed as the means ± SD (n = 3).


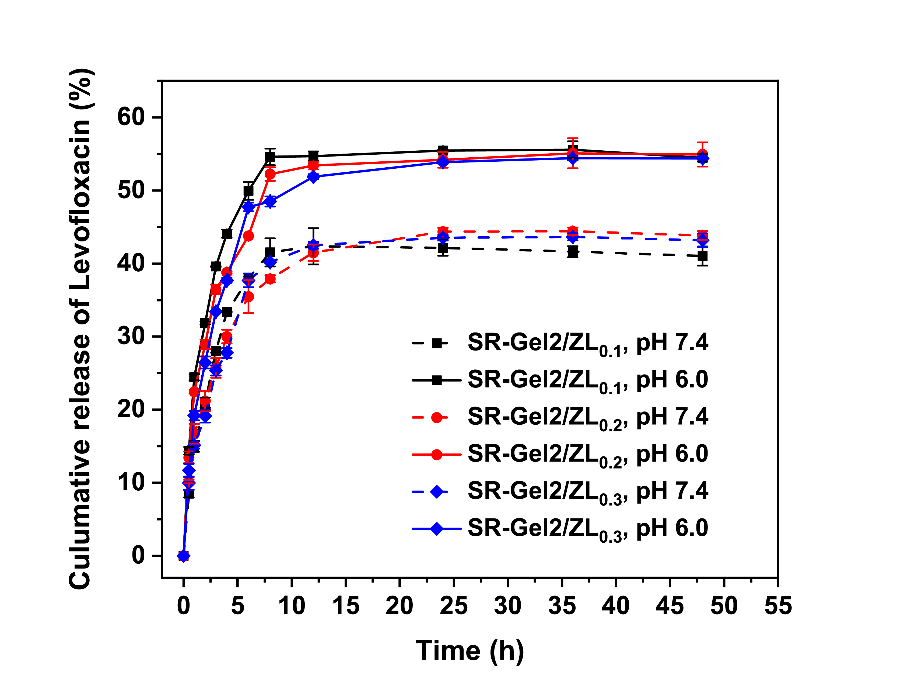


**Figure S18.** The levofloxacin release profiles of SR-Gel/ZL in PBS buffer at pH 7.4 and 6.0. Values are expressed as the means ± SD (n = 3).


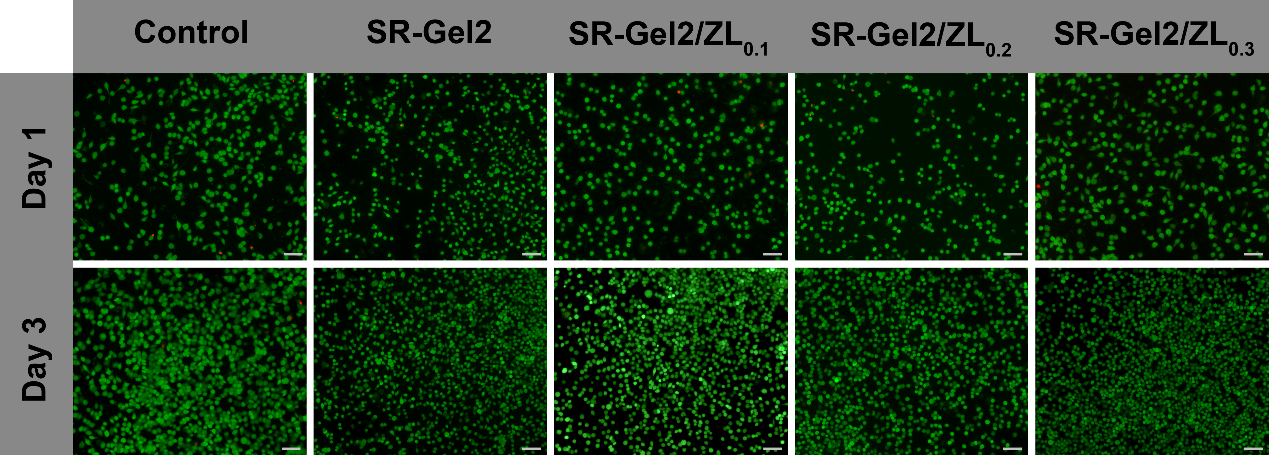


**Figure S19.** Live/dead staining of L929 cells after incubation with the hydrogel extracts for 1, 3 days (scale bar, 100 μm).


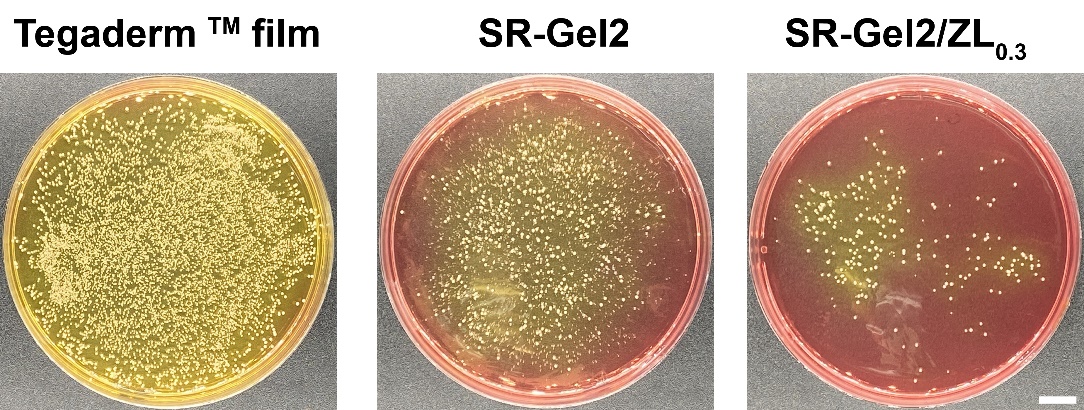


**Figure S20.** Optical images of corresponding bacterial colony-forming units of S. aureus harvested from the infected tissues *in vivo* after treatment with Tegaderm^TM^ film, SR-Gel2 and SR-Gel2/ZL_0.3_ for 2 days (scale bar, 10 mm).


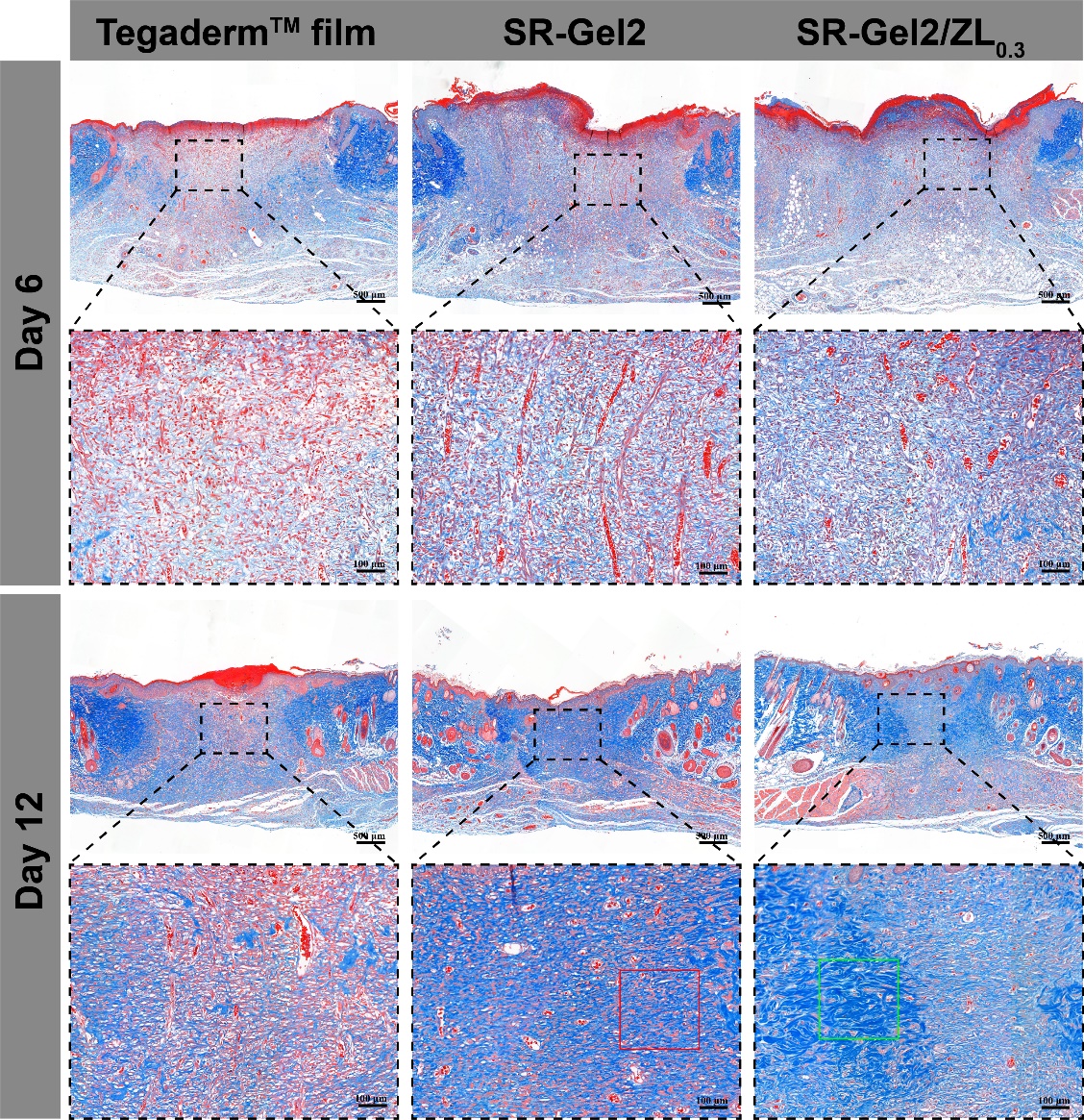


**Figure S21.** Masson’s trichrome staining of tissues collected from wound areas at the 6^th^ and 12^th^ day (scale bar, 100 μm; red box, disorganized collagen fibers; black box, well-organized collagen fibers).


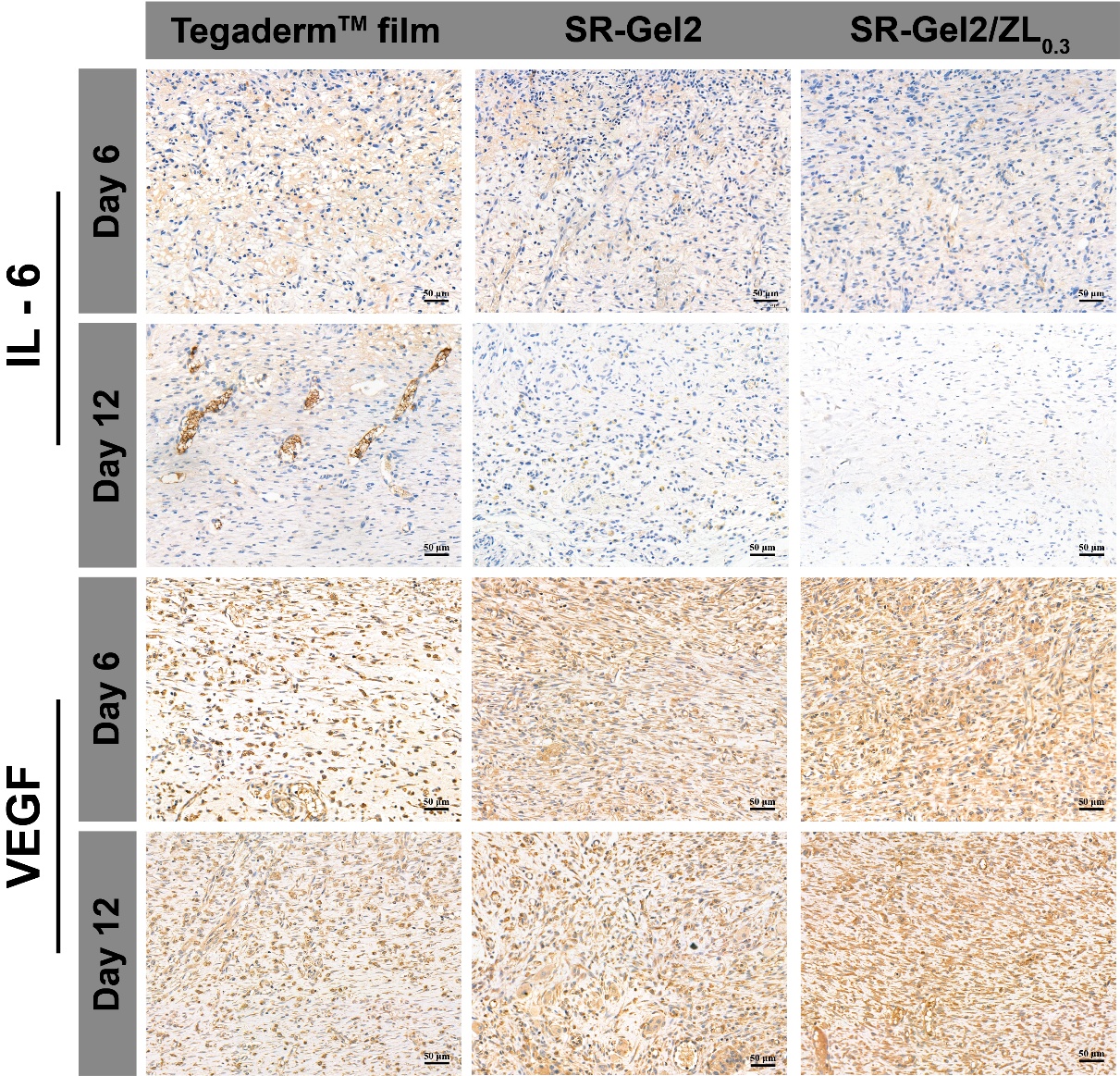


**Figure S22.** Immunohistochemical staining of tissues collected from wound areas at the 6^th^ and 12^th^ day of IL-6 and VEGF.


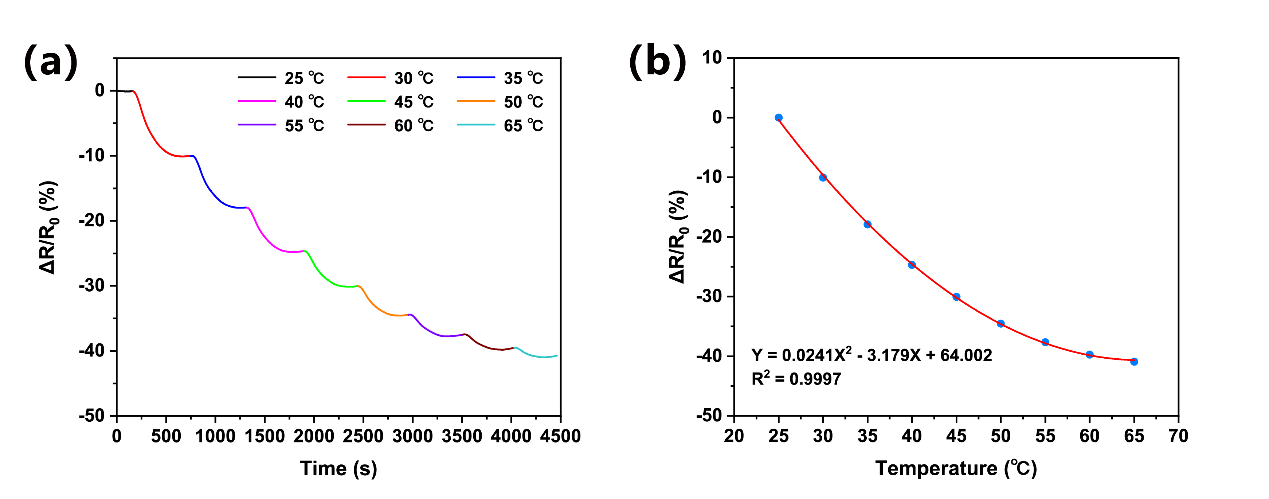


**Figure S23.** (a) The normalized relative resistance curve of SR-Gel2/ZL_0.3_ with temperature change from 25 to 65 ℃. (b) The standard resistance curve of temperature response.


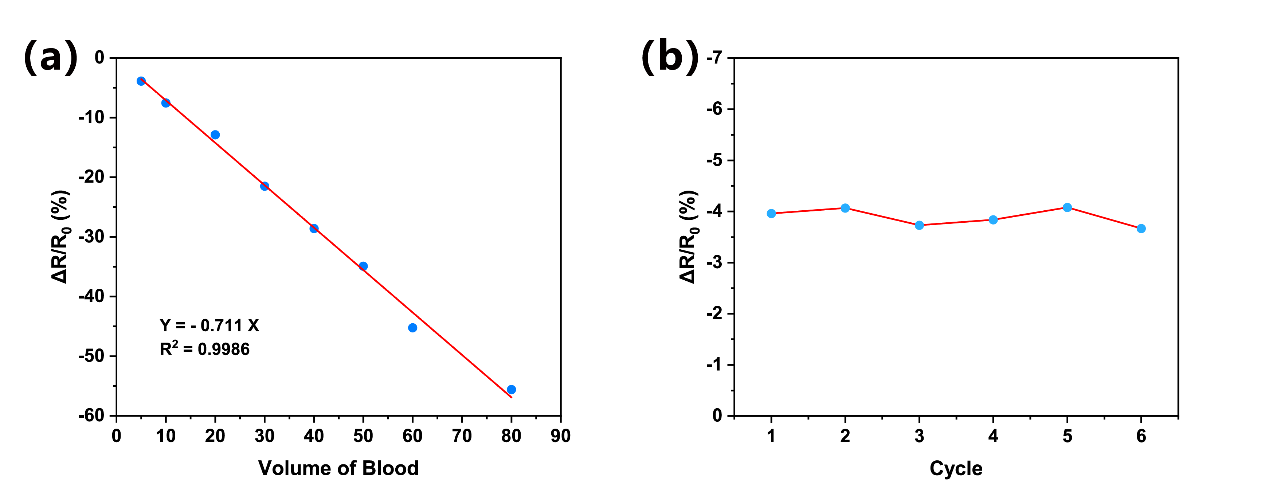


**Figure S24.** (a) The standard resistance curve of blood response. (b) The normalized relative resistance of SR-Gel2/ZL_0.3_ with consecutive response to blood of each 5 μL.


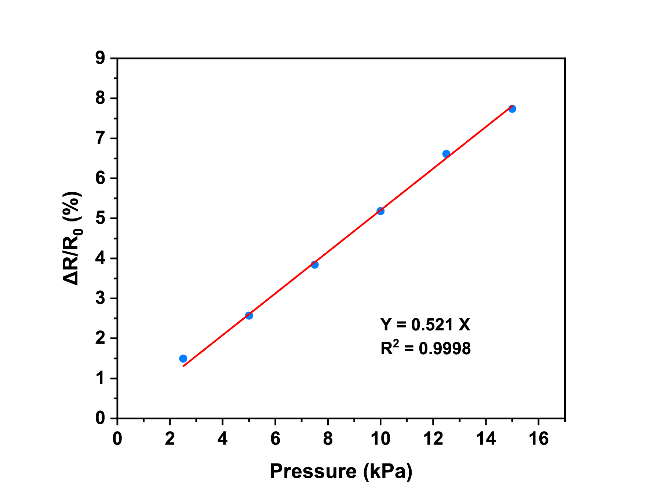


**Figure S25.** The standard resistance curve of pressure response.

**References:**

[1] H. Wang, Y. Wu, C. Cui, J. Yang, W. Liu, Antifouling Super Water Absorbent Supramolecular Polymer Hydrogel as an Artificial Vitreous Body. *Adv. Sci.* **2018**, *5*, 1800711.

[2] K. Kida, M. Okita, K. Fujita, S. Tanaka, Y. Miyake, Formation of high crystalline ZIF-8 in an aqueous solution. *CrystEngComm* **2013**, *15*, 1794.

[3] M. Li, Y. Liang, J. He, H. Zhang, B. Guo, Two-Pronged Strategy of Biomechanically Active and Biochemically Multifunctional Hydrogel Wound Dressing To Accelerate Wound Closure and Wound Healing. *Chem. Mater.* **2020**, *32*, 9937.

[4] Y. Zhu, J. Zhang, J. Yang, C. Pan, T. Xu, L. Zhang, Zwitterionic hydrogels promote skin wound healing. *J. Mater. Chem. B* **2016**, *4*, 5105.

[5] Y. Pan, Y. Liu, G. Zeng, L. Zhao, Z. Lai, Rapid synthesis of zeolitic imidazolate framework-8 (ZIF-8) nanocrystals in an aqueous system. *Chem. Commun.* **2011**, *47*, 2071.

[6] B. Tao, W. Zhao, C. Lin, Z. Yuan, Y. He, L. Lu, M. Chen, Y. Ding, Y. Yang, Z. Xia, K. Cai, Surface modification of titanium implants by ZIF-8@Levo/LBL coating for inhibition of bacterial-associated infection and enhancement of *in vivo* osseointegration. *Chem. Eng. J.* **2020**, *390*, 124621.

[7] a) H. Zheng, Y. Zhang, L. Liu, W. Wan, P. Guo, A. M. Nystrom, X. Zou, One-pot Synthesis of Metal Organic Frameworks with Encapsulated Target Molecules and Their Applications for Controlled Drug Delivery. *J. Am. Chem. Soc.* **2016**, *138*, 962; b) M. Xu, Y. Hu, W. Ding, F. Li, J. Lin, M. Wu, J. Wu, L.-P. Wen, B. Qiu, P. Wei, P. Li, Rationally designed rapamycin-encapsulated ZIF-8 nanosystem for overcoming chemotherapy resistance. *Biomaterials* **2020**, *258*, 120308.
